# An efficient eco-friendly, simple, and green synthesis of some new spiro-N-(4-sulfamoyl-phenyl)-1,3,4-thiadiazole-2-carboxamide derivatives as potential inhibitors of SARS-CoV-2 proteases: drug-likeness, pharmacophore, molecular docking, and DFT exploration

**DOI:** 10.1007/s11030-023-10761-0

**Published:** 2023-11-09

**Authors:** Ahmed M. El-Saghier, Souhaila S. Enaili, Aly Abdou, Asmaa M. Kadry

**Affiliations:** 1 Chemistry Department, Faculty of Science, Sohag University, Sohag, 82524 Egypt; 2 Chemistry Department, Faculty of Science, Al Zawiya University, Al Zawia, Libya

**Keywords:** Green synthesis, Spirooazoles,1,3,4-thiadiazole, Thiocarbohydrazide, 4-Sulfamoylphenylcarboxamide

## Abstract

**Introduction:**

The coronavirus disease 2019 (COVID-19) pandemic has caused a global health crisis. The severe acute respiratory syndrome coronavirus 2 (SARS-CoV-2) is a highly contagious virus that can cause severe respiratory illness. There is no specific treatment for COVID-19, and the development of new drugs is urgently needed.

**Problem statement:**

The SARS-CoV-2 main protease (M^pro^) enzyme is a critical viral enzyme that plays a vital role in viral replication. The inhibition of M^pro^ enzyme can be an effective strategy for developing new COVID-19 drugs.

**Methodology:**

An efficient operationally simple and convenient green synthesis method had been done towards a series of novel spiro-*N*-(4-sulfamoylphenyl)-2-carboxamide derivatives, in ethanol at room temperature in green conditions, up to 90% yield. The molecular structures of the synthesized compounds were verified using spectroscopic methods.The title compounds were subjected to in silico analysis, including Lipinski’s rule and ADMET prediction, in addition to pharmacophore modeling and molecular docking against the active site of SARS-CoV-2 target main protease (M^pro^) enzyme (6LU7). Furthermore, both of the top-ranked compounds (5 and 6) and the standard Nirmatrelvir were subjected to DFT analysis.

**Findings:**

The synthesized compounds exhibited good binding affinity to SARS-CoV-2 Mpro enzyme, with binding energy scores ranging from − 7.33 kcal/mol (compound **6**) and − 7.22kcal/mol (compound **5**) to − 6.54 kcal/mol (compounds **8** and **9**). The top-ranked compounds (**5** and **6**) had lower HOMO–LUMO energy difference (ΔE) than the standard drug Nirmatrelvir. This highlights the potential and relevance of charge transfer at the molecular level.

**Recommendation:**

These findings suggest that the synthesized spiro-N-(4-sulfamoylphenyl)-2-carboxamide derivatives could be potential candidates for COVID-19 drug development. To confirm these drugs' antiviral efficacy in vivo, more research is required. With very little possibility of failure, this proven method could aid in the search for the SARS-CoV-2 pandemic's desperately needed medications.

**Graphical abstract:**

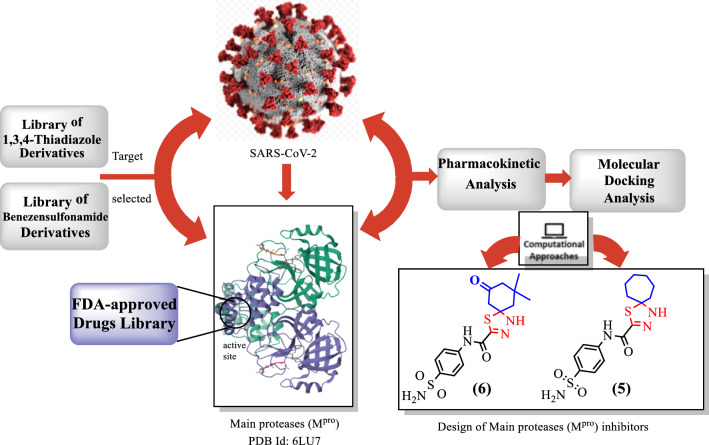

**Supplementary Information:**

The online version contains supplementary material available at 10.1007/s11030-023-10761-0.

## Introduction

Viruses that cause various human illnesses on various organs or tissues and are slow to heal. As a result of genetic alterations, the resistance of viruses is constantly increasing [1]. The coronavirus disease 2019 (COVID-19), a new-brand infectious illness, spread by severe acute respiratory syndrome coronavirus 2 (SARS-CoV-2), traveled quickly in different countries. The public's safety was severely harmed by COVID-19, and in March 2020, the WHO announced it to be a pandemic. Due to the lack of an appropriate medication that can cure the condition of COVID-19 on the market, only a small number of currently available drugs can treat the clinical signs and symptoms of COVID-19. As a result, research into a medicine to treat COVID-19 became a critical requirement in Clinical studies which caught greater curiosity between chemical scientists [2–4]. The 1,3,4-thiadiazole moiety combining with bioisosteres benzenesulfonamide has substantial potential in this effort to create an antiviral activity of a new scaffold because of their pharmacological profile and pharmacokinetic features [5, 6].

**Fig. 1:**
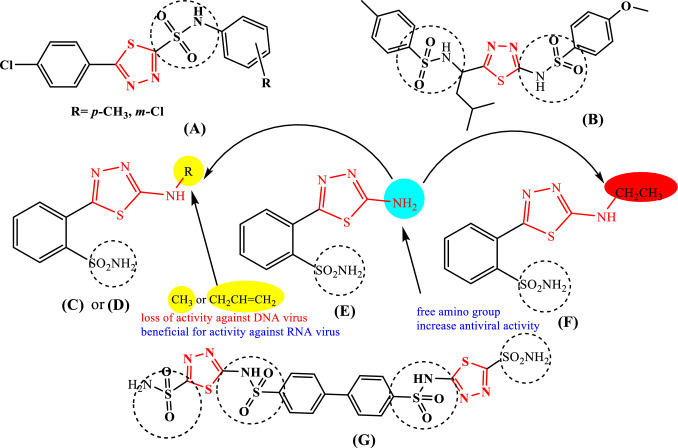
Chemical structure of 1,3,4-thiadiazole derivatives bearing with benzenesulfonamide moiety as antiviral agents

Depending on their molecular structure, mesonic nature, good lipophilicity, highly effective with a less toxic [7, 8], and numerous pharmacological activities, 1,3,4-thiadiazole moieties can engage in a variety of functions as drugs that treat diabetes, cancer, inflammation, spasticity, antivirals, antihypertensive, and bacteria [9–13]. In the literature, Several commercially available drugs have 1,3,4-thiadiazole rings in them, for example, Cefazolin and Cefazedone as cell wall synthesis inhibitors (antibiotics), Megazol as protein and DNA synthesis inhibitor (Antiprotozoal), Methazolamide, and Acetazolamide as carbonic anhydrase inhibitors (Diuretics,) Sulphamethizole as dihydropteroate synthase inhibitor (Antimicrobial), Azetepaan alkylating agent (anti‐Cancer) [5, 14]. Acetazolamide, besaglybuzole (glybuzole), and furidiazine (triafur) were three antiviral drugs that use 1,3,4-thiadiazole hybrid with sulfonamide group [15].The first drug that was widely and consistently utilized as chemotherapeutic and preventative agents against diverse diseases was sulfonamides (sulfa drugs) [16]. Rapid progress in this area has allowed the development of more potent and selective sulfonamide derivatives by linking them to a wide variety of 1,3,4-thiadiazole derivatives (**A**) and (**B**) as antiviral agents, as seen in Fig. 1. Moreover, derivatives of compound **(C)** were prepared. Methyl derivative **(D)** and allyl derivative **(E)** reduced the replication of RNA viruses (Poliovirus 1 and Coxsackie virus B4), while ethyl derivative **(F)** was completely inactive against all viral strains. These results suggest the importance of the side chain for antiviral activity (Fig. 1). Compound **(G)** is the most effective antiviral chemotherapeutic drugs that include these two bioisosteres moieties [17, 18].

**Fig. 2:**
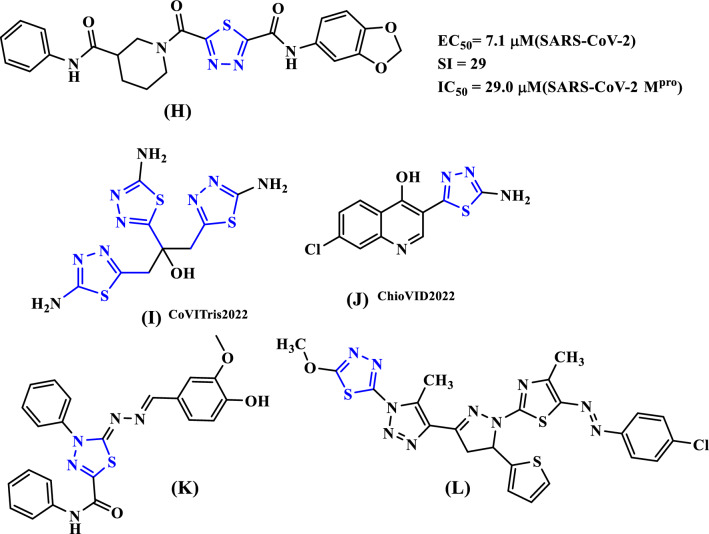
Chemical structure of 1,3,4-thiadiazole derivatives as SARS-Cov-2 inhibitors

Considering the significance of thiadiazole nucleus in the field of medicinal chemistry and the search for novel COVID antiviral drugs, newly design and development compound (**H)** [19] subjected to in silico studies against coronavirus evaluations with EC_50_ = 7.1 mM(SARS-CoV-2) and IC_50_ = 29.0 mM(SARS-CoV-2 M^pro^).The activity of the two 2-aminothiadiazoles, CoViTris2022 **(I)** and ChloViD2022 **(J)** [20], showed significant binding for the coronaviral-2 polymerase/exoribonuclease with the four principal RNA nucleotides. Recent research [21] done on novel thiadiazole derivatives revealed that compound (**K**) exhibited remarkable similarity characteristics and substantial-binding capacities inside the human organs. They also included compound **(L)**, which could potentially treat coronavirus because it had a particularly high binding capacity (-9.1 kcal/mol) toward the target enzyme [22], Fig. 2.

**Fig. 3:**
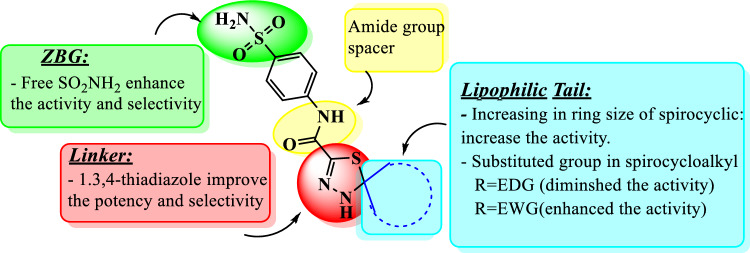
Summary of SAR for spiro-N-(4-sulfamoylphenyl)-1,3,4-thiadiazole-2-carboxamides

As part of our ongoing research, we examine novel heterocyclic nuclei hybridized with bioactive groups that might be employed as catalysts in the manufacture of novel pharmacological models [23–33]. Due to the considerable importance of spirocyclic systems that include a 1,3,4-thiadiazole unit paired with a sulfamoylphenyl moiety [34], in this study, our objective was to design, synthesize, and characterize new series of novel spiro-*N*-(4-sulfamoylphenyl)-2-carboxamide derivatives that are derived from 2-hydrazinyl-N-(4-sulfamoylphenyl)-2-thioxoacetamide. The approved methodology in use offered benefits such as delivering products with high yields and purity while reducing environmental pollution, also without the need for costly catalysis. In addition, a molecular docking analysis of these substances was carried out versus the intended protease. The Drug-likeness analysis of the synthesized compounds was explored in the term of ADMET (Absorption, Distribution, Metabolism, Excretion, and Toxicity) properties.

## Methodology

Thin-layer chromatography (TLC) was employed to track all reactions utilizing percolated dishes of silica gel G/UV-254 with a 0.25 mm thickness (Merck 60F254) and UV light (254 nm/365 nm) enable visualization. The uncorrected Kofeler melting point instrument was used to record all melting points. On an FT-IR spectrophotometer, KBr pellets were used to analyze IR spectra. At Sohag University, ^1^H-NMR and ^13^C-NMR (DMSO-d_6_) spectra were captured at 400 M Hz and 100 MHz, respectively. For ^1^H-NMR data, the following information is provided: chemical shift, integration, and multiplicity (singlet, doublet, triplet, multiplet). Tetramethylsilane (TMS) was selected as the standard for internal measurement, and its chemical shifts (δ) were expressed in parts per million (ppm). TMS (= 0 ppm) or DMSO (= 39.51 ppm) was employed as internal standards for ^13^C-NMR. A Perkin-Elmer CHN analyzer model provided the elemental analyses.

### Synthesis of 2-hydrazinyl-N-(4-sulfamoylphenyl)-2-thioxoacetamide (1)

Synthesized from appropriate 2-chloro-N-sulfamoylphenyl acetamide with morpholine and sulfur, followed by reaction with hydrazine hydrate by a known procedure [35, 36].

#### 2-Hydrazinyl-N-(4-sulfamoylphenyl)-2-thioxoacetamide (1)

Pale yellow solid, Yield (72%). Mp. 185°C. FT-IR (KBr) *ν*_max_ cm^−1^: 3344, 3269, 3175, 3106 (2NH, 2NH_2_), 3095 (CH_-arom_.), 2934 (CH_-aliph_.), 1680 (C=O_amide_, st), and 1375 (S=O, st). ^1^H-NMR (DMSO-*d*_*6*_), δ ppm: 10.40 (s, 1H, NH_amide_), 7.87–7.79 (m, 4H, CH_arom_.); 7.28 (s, 2H, NH_2sulfa_), 3.81 (br, 3H, 3NH); C. F.: C_8_H_10_N_4_O_3_S_2_ M. W: 274.44; Elemental Analysis: Calc; C, 44.32; H, 4.75; N, 16.81; S, 20.09, Found; C, 44.44; H, 4.70; N, 16.78; S, 20.11.

#### Synthesis of spiro-N-(4-sulfamoylphenyl)-1,3,4-thiadiazole-2-carboxamide derivatives (2–12):

**Scheme 1:**
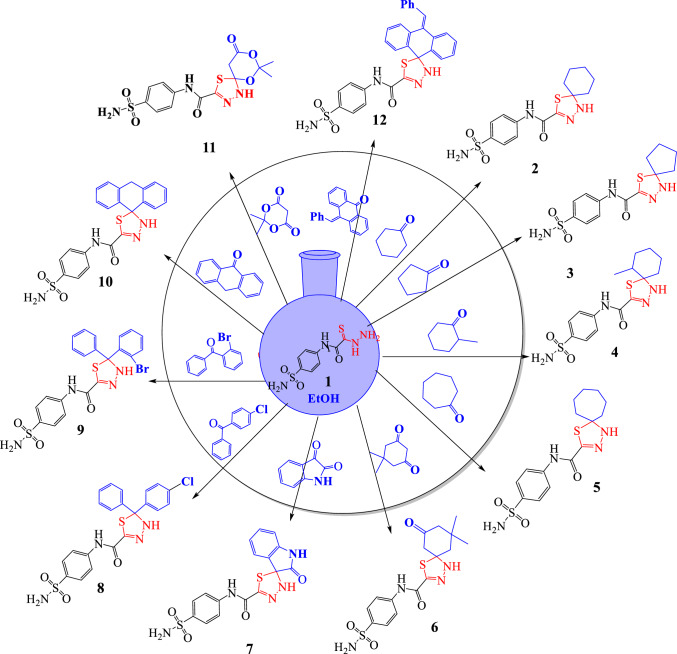
Formation of spiro-N-(4-sulfamoylphenyl)-1,3,4-thiadiazole-2-carboxamides

To a solution of 2-hydrazinyl-N-(4-sulfamoylphenyl)-2-thioxoacetamide **(1)** (0.001 mol) in ethanol (15 mL), (0.001 mol) of ketone derivatives, the reaction mixture was stirring at room temperature for about 3h. The reaction was cooled, and the solid precipitate was collected by filtration and crystalized from ethanol (Scheme1).

#### N-(4-sulfamoylphenyl)-4-thia-1,2-diazaspiro[4.5]dec-2-ene-3-carboxamide (2)

Pale yellow solid, Yield (92%). Mp. 185°C. FT-IR (KBr) *ν*_max_ cm^−1^: 3348, 3314,3223(2NH, NH_2_), 3071(CH_-arom_.), 2928,2857(CH_-aliph_.)1650 (C=O_amide_, st), and 1334(S=O, st). ^1^H-NMR (DMSO-*d*_*6*_), δ ppm: 10.32 (s, 1H, NH amide), 8.57 (s, 1H, NHthiadizole), 7.89–7.74 (m, 4H, CH-arom.); 7.21(s,2H,NH_2_), 2.10–1.24 (m, 10H, 5CH_2_, exchangeable by Dept.), ^13^C-NMR (DMSO-*d*_*6*_), δ ppm: 159.51, 141.69, 139.10, 137.67, 126.90, 120.12, 87.85,39.35, 24.95, 24.20,: C. F.: C_14_H_18_N_4_O_3_S_2_ M. W: 354.44; Elemental Analysis: Calc; C, 47.44; H, 5.12; N, 15.81; S, 18.09, Found; C, 47.44; H, 5.12; N, 15.78; S, 18.11.

### N-(4-Sulfamoylphenyl)-4-thia-1,2-diazaspiro[4.4]non-2-ene-3-carboxamide (3)

Greenish yellow solid, Yield (96%). Mp. 204–206°C. FT-IR (KBr) *ν*_max_ cm^−1^: 3318, 3254 (2NH, NH_2_), 3071(CH-_arom_.), 2971,2883(CH-_aliph_.)1678 (C=O amide, st), and 1313 (S=O, st). ^1^H-NMR (DMSO-*d*_*6*_), δ ppm: 10.32 (s, 1H, NH amide, exchangeable by D_2_O), 8.92 (s, 1H, NHthiadizole, exchangeable by D_2_O), 7.88–7.75 (m, 4H, CH-arom.); 7.27,7.21(s,2H,NH_2_), 2.06–1.63 (m, 8H, 4CH_2,_ exchangeable by Dept.), ^13^C-NMR (DMSO-*d*_6_), δ ppm:159.52, 142.11,141.78, 139.08, 126.73, 119.81, 90.57, 42.22,23.21,: C. F.: C_13_H_16_N_4_O_3_S_2_; M. W.: 340.42; Elemental Analysis: Calc; C, 45.87; H, 4.74; N, 16.46; S, 18.84, Found; C, 45.90; H, 4.71; N, 16.43; S, 18.80.

#### 6-Methyl-N-(4-sulfamoylphenyl)-4-thia-1,2-diazaspiro[4.5]dec-2-ene-3-carboxamide (4)

Brownish yellow solid, Yield (90%). Mp. 199–201 °C. FT-IR (KBr) *ν*_max_ cm^−1^: 3350, 3310,3231(2NH, NH_2_), 3101(CH-arom.), 2963,2931,2859(CH-aliph.), 1648 (C=O amide, st), and 1336(S=O, st). ^1^H-NMR (DMSO-*d*_*6*_), δ ppm: 10.32 (s, 1H, NH amide), 8.76(s, 1H, NH, thiadizole), 7.89–7.73 (m, 4H, CH-arom.); 7.24 (br, 2H, NH_2_); 2.20(m,1H, CH), 1.72–1.28 (m, 8H, 4CH_2_),0.98 (d, 3H, CH_3_) ^13^C-NMR (DMSO-*d*_*6*_), δ ppm: 159.52, 141.88, 139.00, 135.81, 126.88, 120.03, 93.30, 41.75, 32.74, 25.18, 24.16 and 17.89.; C.F.: C_15_H_20_N_4_O_3_S_2_; M. W.: 368.47; Elemental Analysis: calc;C, 48.90; H, 5.47; N, 15.21;S, 17.40.; Found; C, 48.91; H, 5.46; N, 15.19;S, 17.37.

#### N-(4-Sulfamoylphenyl)-4-thia-1,2-diazaspiro[4.6]undec-2-ene-3-carboxamide (5)

Deep yellow solid, Yield (91%). Mp. 214–216 °C. FT-IR (KBr) *ν*_max_ cm^−1^: 3427, 3324,3241(2NH, NH_2_), 3071(CH-arom.), 2931,2895,2855(CH-aliph.),1676 (C=O amide, st), and 1286(S=O, st). ^1^H-NMR (DMSO-*d*_*6*_), δ ppm: 10.37 (s, 1H, NH amide), 8.90 (s, 1H, NH thiadiazole), 7.89–7.73 (m, 4H, CH-arom.); 7.25(br, 2H, NH_2_), 2.18–1.35 (m, 12H, 6CH_2_), ^13^C-NMR (DMSO-*d*_*6*_), δ ppm: 159.44, 142.02, 139.12, 130.45, 129.78, 127.05, 120.12, 90.74, 43.76, 42.28, 30.31, 28.12, 24.36,22.93.; C.F.: C_15_H_20_N_4_O_3_S_2_; M. W.: 368.47; Elemental Analysis: calc. C, 48.90; H, 5.47; N, 15.21; S, 17.40; Found. C, 48.89; H, 5.50; N, 15.11; S, 17.39.

#### 7,7-Dimethyl-9-oxo-N-(4-sulfamoylphenyl)-4-thia-1,2-diazaspiro[4.5]dec-2-ene-3-carboxamide (6)

Greenish yellow solid, Yield (90%). Mp. *d* > 300 °C. FT-IR (KBr) *ν*_max_ cm^−1^: 3344, 3271,3179 (2NH, NH_2_), 3109 (CH-arom.), 2937 (CH-aliph.), 1722 (C=O dimedone, st), 1682 (C=O amide, st), and 1286(S=O, st). ^1^H-NMR (DMSO-*d*_*6*_), δ ppm: 10.50 (s, 1H, NH amide), 9.46 (s, 1H, NH, thiadiazole), 7.95–7.86 (m, H, CH-arom.); 7.31 (s, 2H, NH_2_); 4.52(s,2H,CH_2_), 1.69–1.65 (m, 4H, 2CH_2_), 1.06–0.96(m, 6H, CH_3_); ^13^C-NMR (DMSO-*d*_*6*_), δ ppm: 168.14, 159.00, 140.80, 140.12,133.25,127.06, 90.89, 61.22, 57.11, 23.71 and 21.78.; C.F.: C_16_H_20_N_4_O_4_S_2_; M. W.: 396.48.Elemental Analysis: calc.; C, 48.47; H, 5.08; N, 14.13;S, 16.17; Found.; C, 48.44; H, 5.00; N, 14.11;S, 16.15.

#### 2-Oxo-N-(4-sulfamoylphenyl)-3'H-spiro[indoline-3,2'-[1,3,4]thiadiazole]-5'-carboxamide (7)

Orang solid, Yield (98%). Mp. 255–257 °C. FT-IR (KBr) *ν*_max_ cm^−1^: 3315, 3281,3220(2NH, NH_2_), 3104, 3054(CH-arom.), 2903(CH-aliph.), 1714 (C=O isatin, st), 1677 (C=O amide, st), and 1271 (S=O, st). ^1^H-NMR (DMSO-*d*_*6*_), δ ppm: 10.60, 10.54 (s, 2H, 2NH amide, exchangeable by D_2_O), 9.63 (s, 1H, NH, thiadiazole, exchangeable by D_2_O) 7.93–6.87 (m, 8H, CH-arom.); 7.23 (s, 2H, NH_2,_exchangeable by D_2_O);^13^C-NMR (DMSO-*d*_*6*_), δ ppm:175.63, 158.47, 141.68, 139.36, 137.43, 131.53, 129.42, 126.97, 126.39, 123.52, 120.35, 110.78 and 80.54.; C.F.: C_16_H_13_N_5_O_4_S_2_, M.W.: 403.Elemental Analysis: calc. C, 47.64; H, 3.25; N, 17.36; S, 15.89; Found, C, 47.66; H, 3.21; N, 17.30; S, 15.90.

#### 5-(4-Chlorophenyl)-5-phenyl-N-(4-sulfamoylphenyl)-4,5-dihydro-1,3,4-thiadiazole-2-carboxamide (8).

Pale yellow, Yield (91%). Mp. > 300 °C. FT-IR (KBr) *ν*_max_ cm^−1^: 3345, 3273, 3249(2NH, NH_2_), 3110(CH-arom.), 2935(CH-aliph.)1680 (C=O amide, st), and 1286(S=O, st), 652(C–Cl). ^1^H-NMR (DMSO-*d*_*6*_), δ ppm: 13.09, (s, 1H, NH amide), 10.47 (s, 1H, NH, thiadizole), 7.95–7.28 (m, 13H, CH-arom.); 7.23 (s, 2H, NH_2_); ^13^C-NMR (DMSO-*d*_*6*_), δ ppm: 159.19, 140.76, 140.09, 129.24, 128.95, 128.68, 127.08, 126.95, 120.49 and 56.72.; C.F; C_21_H_17_ClN_4_O_3_S_2_, M. W.: 472.96.Elemental Analysis: C, 53.33; H, 3.62; Cl, 7.50; N, 11.85; S, 13.56; Found; C, 53.36; H, 3.60; Cl, 7.48; N, 11.75; S, 13.45.

#### 5-(2-Bromophenyl)-5-phenyl-N-(4-sulfamoylphenyl)-4,5-dihydro-1,3,4-thiadiazole-2-carboxamide (9).

Pale yellow, Yield (92%). Mp. 224–226 °C. FT-IR (KBr) *ν*_max_ cm^−1^: 3345, 3278, 3244(2NH, NH_2_), 3089, 3062 (CH-arom.), 2913 (CH-aliph.), 1679 (C=O amide, st), and 1285(S=O, st), 688 (C–Br). ^1^H-NMR (DMSO-*d*_*6*_), δ ppm: 13.86 (s, 1H, NH amide), 10.18 (s, 1H, NHthiadiazol), 7.96–7.32 (m, 13H, CH-arom.); 7.25 (br, 2H, NH_2_); ^13^C-NMR (DMSO-*d*_*6*_*)*, δ ppm: 159.08, 141.21, 140.79, 139.86, 139.17, 137.40, 136.53, 133.36, 132.03, 131.45, 130.03, 129.32, 127.15, 120.45,56.52C.F.: C_21_H_17_BrN_4_O_3_S_2_; M. W.: 517.42; Elemental Analysis: calc; C, 48.75; H, 3.31; Br, 15.44; N, 10.83; S, 12.39:; found: C, 48.76; H, 3.29; Br, 15.39; N, 10.80; S, 12.40.

#### N-(4-Sulfamoylphenyl)-3'H,10H-spiro[anthracene-9,2'-[1,3,4]thiadiazole]-5'-carboxamide (10)

Brownish yellow solid, Yield (95%). Mp. 220–222 °C. FT-IR (KBr) *ν*_max_ cm^−1^: 3340, 3267, 3239 (2NH, NH_2_), 3098 (CH-arom.), 2964 (CH-aliph.), 1681 (C=O amide, st), and 1329(S=O, st). ^1^H-NMR (DMSO-*d*_*6*_), δ ppm: 10.51 (s, 1H, NH amide), 10.36 (s, 1H, NHthaidizole), 8.06–7.78 (m, 12H, CH-arom.); 7.24 (br, 2H, NH_2_); 4.20 (s,2H,CH_2_); ^13^C-NMR (DMSO-*d*_*6*_), δ ppm: 166.05,142.21, 141.70, 141.05, 140.39, 139.09, 127.06,121.53,121.02, 120.61, 120.15, 119.64, 92.48,27.92.; C. F.: C_22_H_18_N_4_O_3_S_2_; M. W.: 450.53.Elemental Analysis: calc; C, 58.65; H, 4.03; N, 12.44; S, 14.23; Found; C, 58.67; H, 4.00; N, 12.31; S, 14.22.

#### 7,7-dimethyl-9-oxo-N-(4-sulfamoylphenyl)-6,8-dioxa-4-thia-1,2-diazaspiro[4.5]dec-2-ene-3-carboxamide (11)

Pale brawn solid, Yield (90%). Mp. > 300°C. FT-IR (KBr) *δ*_max_ cm^−1^: 3378, 3342, 3239(2NH, NH_2_), 3093(CH-arom.), 2963, 2930(CH-aliph.), 1701(C=O ring, st.),1680(C=O amide, st), and 1317(S=O, st). ^1^H-NMR (DMSO-*d*_*6*_), δ ppm: 11.08 (s, 1H, NH amide), 10.36 (s, 1H, NH thaidizole), 7.94–7.78 (m, 4H, CH-arom.), 7.21(br, 2H, NH_2_), 4.18–3.65(m, 2H, CH_2_), 1.12–1.07(m, 6H, 2CH_3_); C.F.: C_20_H_14_N_4_O_3_S_2_. M. W.: 422.48. Elemental Analysis: calc. C, 56.86; H, 3.34; N, 13.26; S, 15.18,; Found; C, 56.88; H, 3.33; N, 13.18; S, 15.15.

#### 10-Benzylidene-N-(4-sulfamoylphenyl)-3'H,10H-spiro[anthracene-9,2'-[1,3,4]thiadiazole]-5'-carboxamide (12)

Deep brawn solid, Yield (93%). Mp. 232–234 °C. FT-IR (KBr) *ν*_max_ cm^−1^: 3350, 3259,3204(2NH, NH_2_), 3106(CH-arom.), 2928(CH-aliph.)1674 (C=O amide, st), and 1313 (S=O, st). ^1^H-NMR (DMSO-*d*_*6*_), δ ppm: 10.47 (s, 1H, NH amide), 9.91 (s, 1H,), 8.23–7.03 (m, 18H, CH-arom.).); 6.45 (br, 2H, NH_2_); ^13^C-NMR (DMSO-d_6_), δ ppm: 159.07, 140.84, 140.06, 135.56, 135.04, 133.28, 133.05, 132.63,130.55, 129.88,128.15, 127.23, 126.04, 124.11, 123.63, 120.48, 53.08.; C.F.: C_29_H_22_N_4_O_3_S_2_; C. F.: 538.64.Elemental Analysis: calc.: C, 64.67; H, 4.12; N, 10.40; S, 11.90; Found: C, 64.77; H, 4.08; N, 10.22; S, 11.85.

### Drug-likeness analysis

The drug-likeness analysis, including Lipinski’s rule of five as well as ADMET, an abbreviation denoting the fundamental processes of absorption, distribution, metabolism, and excretion, as well as toxicity, prediction of the title compounds were calculated in ADMET lab 2.0 tool (https://admetmesh.scbdd.com/) [37].

### Pharmacophore investigation

The pharmacophore generation protocol was performed using MOE on a training set database consisting of ten FDA-approved drugs for COVID-19 therapy: Chloroquine, Cycloheximide, Emetine, Exalamide, Hycanthone, Lycorine, Promazin, Propranalol, Trilorene, and Zoxazolamine, Figure S38. The database compounds were subjected to energy minimization and flexible alignment before performing the pharmacophore search. The ‘feature mapping’ protocol was applied to identify the common features among the database compounds [38].

The validation of the pharmacophore model was done using the internal validation method by using the training set (the molecules used to generate the model) and by using two active Nirmatrelvir and Ritonavir drugs, to test the model’s performance.

### Molecular docking investigation

The investigation of the binding power of the aforementioned compounds to the SARS-CoV-2 enzyme protease (6LU7) was conducted through the utilization of molecular docking strategy [39, 40]. The generation of the three-dimensional models of the title compounds was done utilizing the builder user interface of MOE [41]. The ligands were constructed through the generation of new database, followed by the implementation of protonate 3D, partial charge assignment, and energy minimization process on the respective title compounds [42, 43]. The ligands have been saved in an MDB file format to facilitate the docking calculation [44, 45].The crystallographic arrangement of the SARS-CoV-2 protease enzyme (6LU7) was acquired from the Protein Data Bank (PDB) repository, accessible at https://www.rcsb.org/structure/6LU7 [46]. Then, the protein underwent protonation and acquired a charge through utilization of the protonate 3D module within the MOE software. The bond and atom types were thoroughly examined and duly assigned, considering the hydrogen atoms, receptor, and atom potentials [47, 48]. The identification of the active site of the enzyme was accomplished through the utilization of the MOE Alpha Site Finder. The conformation of the active site was optimized to incorporate the residues that engage in interactions with the receptor [49, 50]. Docking experiments were conducted to evaluate the binding free energy of the inhibitor-protein complex. The London dispersion-based free energy (dG) scoring function was employed to evaluate the scoring metrics in molecular docking investigations [51]. The exported docking poses and interaction parameters were utilized to rank the inhibitory activity based on scoring (abbreviated as S, kcal/mol) and to analyze the interaction features. The Nirmatrelvir as standard drug was used as standard for docking studies and for comparison of docking scores with the investigated compounds.

The docking procedure was validated through the utilization of the re-docking and overlaying methodology. The indigenous ligand derived from the 6LU7 structure was isolated and subsequently repositioned within the active site employing an identical docking methodology [52].

### Density functional theory (DFT) analysis

Density functional theory (DFT) analyses play an essential role in the computation of molecular orbital characteristics [53, 54]. In this framework, the top two compounds from the screening process (**5**, and **6**) underwent a structure-based DFT analysis utilizing B3LYP [55, 56] and a 6-31 g + (d,p) [57] basis set using Gaussian 09w [58]. Comparative research between the highest occupied molecular orbital (HOMO) and lowest unoccupied molecular orbital (LUMO) energies was performed [59–61].

## Results and discussion

### Chemistry

In continuation of our work in the synthesis of novel spiro-heterocycles [62–65], we prepared in this article a new series of spiro-*N*-(4-sulfamoylphenyl)-2-carboxamide derivatives containing a 1,3,4-thiadiazole unit, in a new method, smooth way, one-pot reaction, saving energy (at room temperature), low cost (without catalyst), short period, green solvent, no requirements for toxic chemicals, and high yield which achieve the green synthesis rules. In the present wor, we used the reported method by Yarovenko et al. (2003) for synthesis of 2-hydrazinyl-N-(4-sulfamoylphenyl)-2-thioxoacetamide **(1)** that involves the reaction of 2-chloro-N-sulfamoylphenyl acetamide with morpholine and sulfur, followed by reaction with hydrazine hydrate as illustrated in (Eq. 1.) and used it as a novel building block nucleus in further preparations.

1 $$ {\text{preparation method of 2}} - {\text{hydrazinyl}} - {\text{N}} - \left( {{4} - {\text{sulfamoylphenyl}}} \right) - {2} - {\text{thioxoacetamide }}({\mathbf{1}}). $$

The desired compounds synthesized using new method by stirring2-hydrazinyl-N-(4-sulfamoylphenyl)-2-thioxoacetamide (**1)** under green condition in ethanol at room temperature with a variety of ketones namely, cyclohexanone, cyclopetanone, 2-methylcyclohexanone, cycloheptanone, dimedone, isatin, anthracen-9(10H)-one, acetonaphthaylene-1,2-dione, 10-benzylideneanthracene-9(*10H*)-one, and acyclic ketones, namely, 4-chlorobenzophenone and 2-bromobenzophenone to afford new spiro-N-(4-sulfamoylphenyl)-2-carboxamide derivatives **2–12** (Scheme1).

**Scheme 2:**
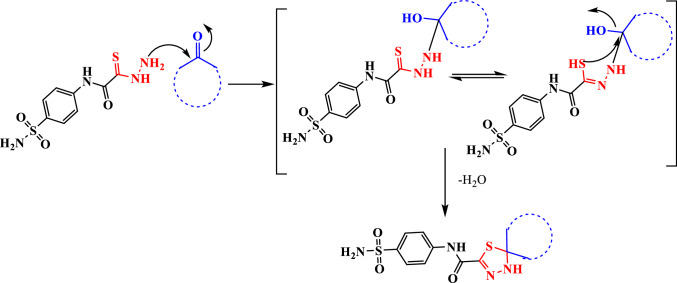
Reaction mechanism for Spiro-N-(4-sulfamoylphenyl)-1,3,4-thiadiazole-2-carboxamides

The reaction mechanism was assumed via a nucleophilic attack of amino group of thiocarbohydrazide**1** at the carbonyl group of ketone followed via a nucleophilic attack of hydrazine group at the carbonyl carbon of ketone to afford thiohydrazone followed by a nucleophilic attack of thiol group at the same carbonyl group of ketone with elimination of water and cyclization (Scheme 2).

The formation of spiro-N-(4-sulfamoylphenyl)-2-carboxamide derivatives **2–12** was validated from its physical and spectral data. In the FT-IR spectrum, NH, NH_2_ stretching band at 3370–3180 cm^−1^, C=H (aromatic) stretching band at 3070–3036 cm^−1^, CH/CH_2_(aliphatic) stretching band of cycloalkane at 2980–2970 cm^−1^ and new bands of C=O for compound **7,6, and 11** at 1710, 1690, and 1701 cm^−1^, respectively. In addition, it was demonstrated that the creation of the spiro system had progressed as evidenced by the removal of the C=S stretching band at 1280–1160 cm^−1^ and the appearance of a C–S–C stretching band at 748 cm-^1^. In the ^1^H-NMR spectra, the signals at 10.50–9.90 (s, NH-thidiazole), 9.30–9.00 (s, NH– C=O), 8.30–7.20 (m, CH–aromatic), 7.10–6.30 (s, 2H, NH_2_), 4.60–3.70 (m, CH_2_- C=O), and 2.90–1.10 (m, CH_2_-cyclic) confirmed the formation of compounds**2-12**.

The formation of spiro compounds **2–12** was proved by a clear band at 80.90–79.20 ppm in the ^13^C-NMR. Finally, the DEPT-135 of compounds **2,3** obviously distinguished characteristic negative signals of three CH_2_ groups of cyclohexane ring at 24.20, 24.93, 39.35 ppm and two CH_2_ groups of cyclopentane ring at 23.07, 42.09 ppm, it showed two CH groups of aromatic rings with a positive phase at 120.12, 126.89 and 120.15, 127.07 ppm, respectively.

### Structure activity relationship (SAR):

The results from our data revealed that spiro-N-(4-sulfamoylphenyl)-1,3,4-thiadiazole-2-carboxamides has potent activity as antiviral agent. In actual, the slight modification of desired compounds led to dramatic change in their activity and revealed that

- By increasing the ring size of spiro-cycloalkyl lipophilic tail, the activity of compounds **2–5** increased. So, compound **5** showed the highest binding energy between them with binding score − 7.22. Moreover, the presence of methyl group (electron donating group (EDG)) as a substituted in cyclohexanone ring, decreases it.
- According compound **6**, The presence of carbonyl group (electron withdrawing group (EWG)) inside cyclohexanone enhanced the binding energy and illustrated the best binding score − 7.33.
- Methylene group in compound **10** obstructed the resonance process in phenyl groups, so decrease the activity. But, replacing two protons in the methylene with benzylidene group (EWG)in compound **12** led to a relative increase in activity as a result of the appearance of conjugation with adjacent benzene ring.
- The presence of sulfonamide group which considered one of compounds incorporating sulfur-based zinc-binding groups (ZBGs) and responsible for improve the activity and selectivity. Moreover, 1,3,4-thiadiazole was essential in enhancing the potency (Fig. 3).

### Drug-likeness and ADMET properties

In our investigation of drug-likeness, our primary emphasis was placed on Lipinski's rule of five (RO5) [66, 67]. According to it, in order for a substance to exhibit efficacy as a small molecule pharmaceutical candidate, it must adhere to the subsequent set of criteria: the compound's molecular weight should not exceed 500, the octanol–water partition coefficient (Log P) should not surpass 5, the number of hydrogen (H) bond donors should not exceed 5, and the number of hydrogen bond acceptors should not exceed 10. The compound was excluded from the active pool due to its non-compliance with the rule. All the compounds investigated in this study demonstrate adherence to the rule, as indicated in the Supporting Information, Table (S1). This observation implies that these compounds possess characteristics that are consistent with drug-like properties.

**Fig. 4:**
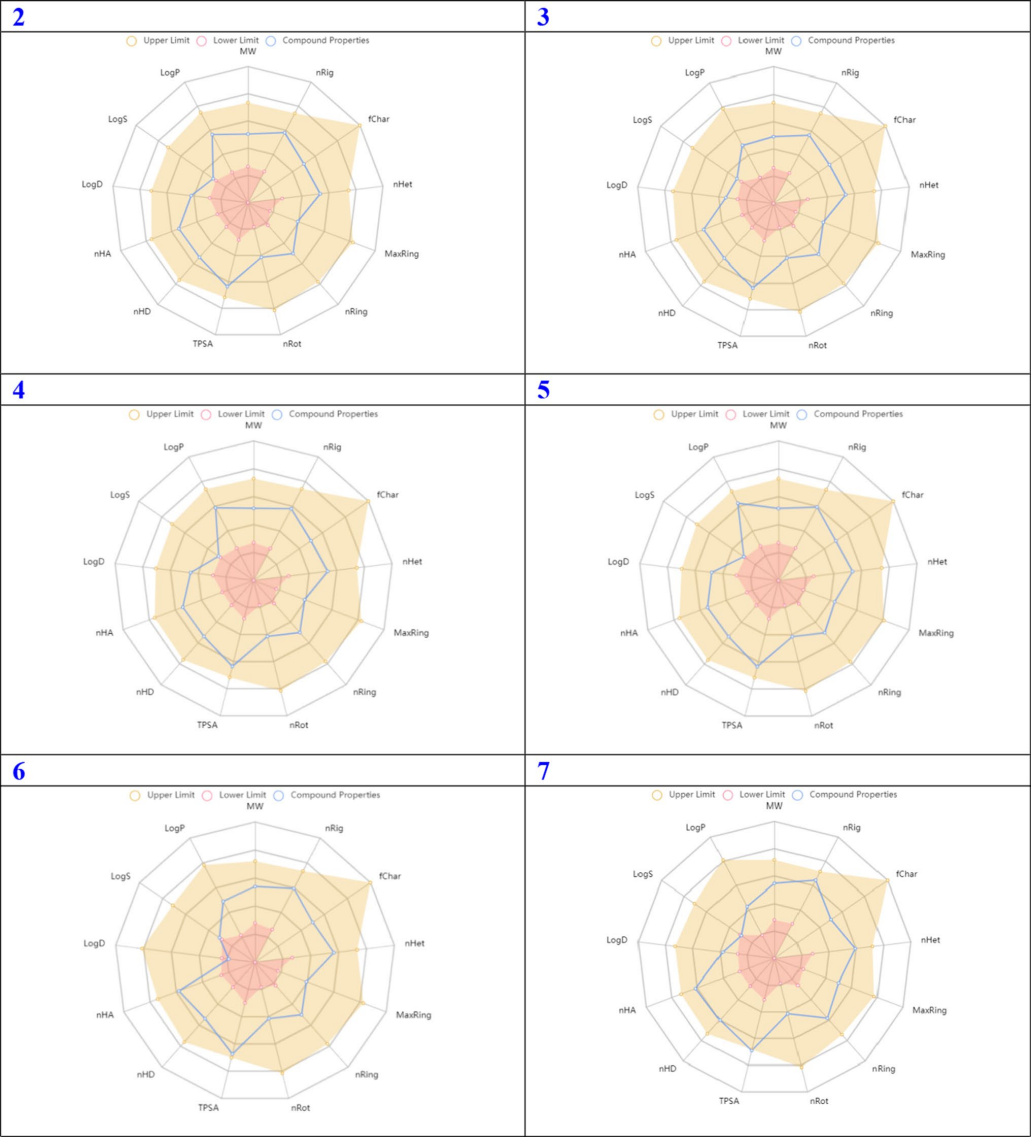

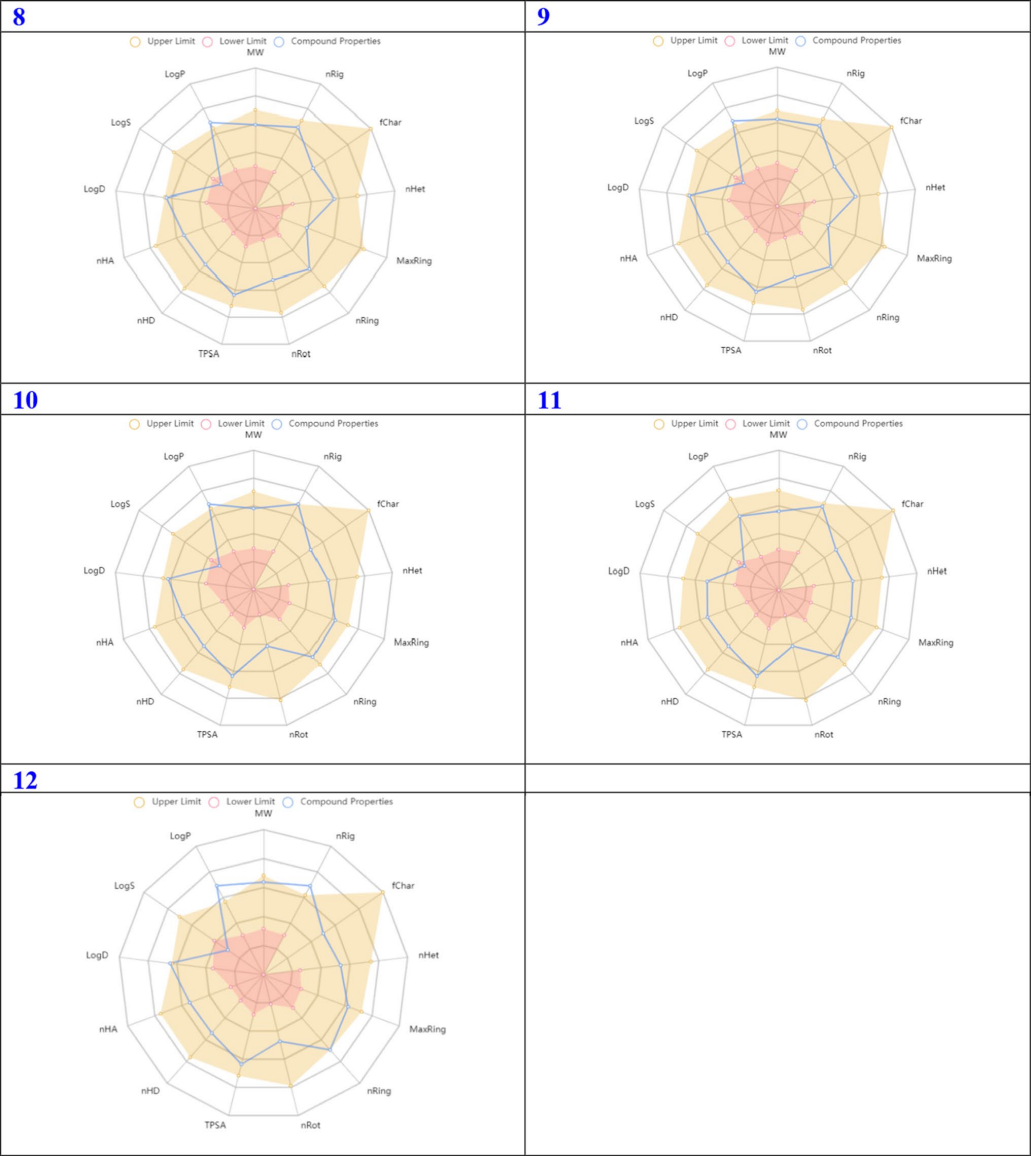
Predicted ADMET properties of the title compounds

In order for a compound to be deemed a promising candidate for drug selection, it is imperative that it exhibits a notable degree of biochemical activity, coupled with a propitious profile in terms of ADMET. In recent times, a plethora of software and online platforms have emerged with the purpose of forecasting the ADMET properties of potential antitumor drug candidates. In the past year, the scientific community has witnessed the emergence of ADMETlab 2.0, a meticulously redesigned online platform dedicated to the accurate prediction of ADMET properties. This cutting-edge web service has undergone extensive reengineering to ensure its optimal performance and reliability. Notably, it has now become commercially available, although it is worth mentioning that it continues to be offered to users without any financial burden [37]. A considerable fraction of scientists is utilizing this platform to discover innovative antineoplastic agents. In accordance with scientific protocol, the utilization of ADMETlab 2.0 was employed to make predictions regarding the ADMET properties of the aforementioned compounds. The predicted ADMET (Absorption, Distribution, Metabolism, Excretion, and Toxicity) properties of the tested compounds are presented in Fig. 4. These properties included the molecular weight, denoted as Mw, nRig, which represents the quantity of rigid bonds present. The term "fChar" represents the formal charge, where nHet represents the number of heteroatoms present. MaxRing: The maximum number of atoms present in the largest ring within the molecular structure. The parameter nRing represents the quantity of rings present in the system under consideration. The term "nRot" represents the quantity of rotatable bonds present in the system. TPSA, which stands for Topological Polar Surface Area. The nHD which represents the number of hydrogen bond donors, nHA represents the count of hydrogen bond acceptors. logD refers to the logarithm of the partition coefficient (logP) at a physiological pH of 7.4. logs that represent the logarithm of the aqueous solubility. The logP refers to the logarithm of the partition coefficient between octanol and water. As depicted in Fig. 4, it is observed that all the examined samples fall within the designated 'upper limit' region (highlighted in yellow in Fig. 4. Consequently, it can be inferred that all the properties exhibited satisfactory performance. The computational analyses have revealed that these compounds demonstrate exceptional ADMET properties. The comprehensive statistical analysis of ADMET calculations can be readily accessed in the Supporting Information, specifically in Table S1.

### Pharmacophore analysis

**Fig. 5:**
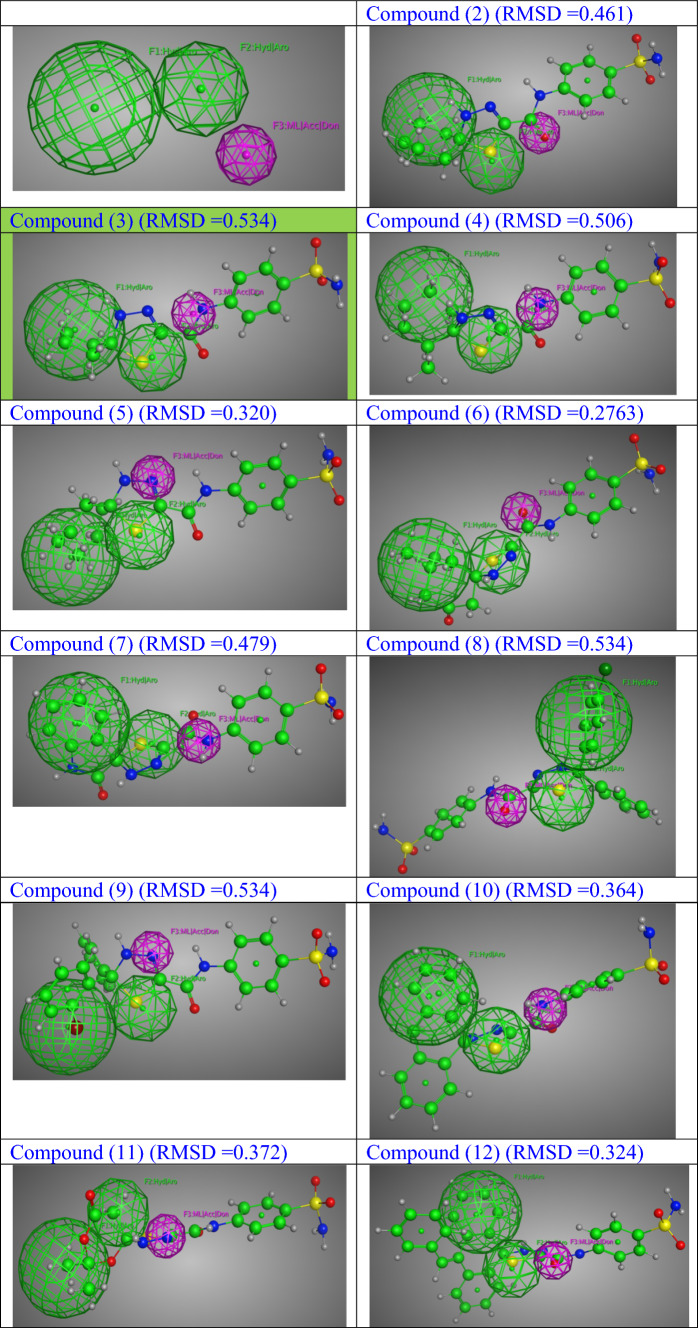
The developed pharmacophore model, where Hyd: Hydrophobic, Aro: Aromatic center, Acc: H-bond acceptor, Don: H-bond acceptor, ML: Metal Ligator, and the chosen molecular entities derived from the pharmacophoric characteristics for the compounds under investigation (2, 3, 4, 5, 6, 7, 8, 9, 10, 11, 12), with their (RMSD)

The pharmacophore model was obtained through the alignment of the molecular structures of the ten FDA-approved active compounds against COVID-19, which comprise the training set. The alignment revealed a favorable fit [68, 69], as depicted in Supplementary Information Figure S38. The pharmacophore model was composed of three indispensable characteristics: Feature F1 exhibits hydrophobic properties due to its chemical structure, characterized by the presence of hydrophobic groups. Similarly, Feature F2 also displays hydrophobic characteristics owing to its chemical composition. On the other hand, Feature F3 is classified as a metal legator, demonstrating both electron-accepting and donating capabilities. These molecular properties are visually represented in Fig. 5. The three characteristics were employed to scrutinize the assessed database (2, 3, 4, 5, 6, 7, 8, 9,10, 11, 12) with the aim of discerning plausible inhibitors for COVID-19. All the compounds (2, 3, 4, 5, 6, 7, 8, 9, 10, 11, 12) subjected to examination were found to satisfy the criteria outlined in the pharmacophore model, as depicted in Fig. 5. Henceforth, it has been observed that all the compounds subjected to experimentation, namely 2, 3, 4, 5, 6, 7, 8, 9, 10, 11, and 12, have exhibited structurally favorable attributes for the inhibition of enzymes as well as the inhibition of COVID-19. The quantification of the deviation from the pharmacophore model was determined through the utilization of the root-mean-square deviation (RMSD) technique, which involved superimposing the molecular structure onto the pharmacophore model (as depicted in Fig. 5). Based on RMSD values, the reactivity order of the tested compounds can be arranged as follows: Compound **6** (RMSD = 0.2763) exhibits the highest reactivity, followed by Compound **5** (RMSD = 0.32), Compound **12** (RMSD = 0.324), Compound **10** (RMSD = 0.364), Compound **11** (RMSD = 0.372), Compound **2** (RMSD = 0.461), Compound **7** (RMSD = 0.479), Compound **4** (RMSD = 0.506), Compound **3** (RMSD = 0.534), Compound **8** (RMSD = 0.534), and Compound **9** (RMSD = 0.534).

The validation of the pharmacophore model was done using the internal validation method by using the training set (the molecules used to generate the model) and also by using two active Nirmatrelvir and Ritonavir drugs, to test the model’s performance. The results, Figure S39, showed good performance of the pharmacophore model with lower values of RMSD.

### Molecular docking

The evaluation of pharmacological effectiveness for novel compounds typically involves the investigation of their susceptibility to interactions with primary goals, namely proteins [70, 71]. In this investigation, the technique of Molecular docking was employed to elucidate the intricate interplay among the compounds under scrutiny and the selected protein. Molecular docking is a computational methodology employed to ascertain the anticipated biological efficacy of pharmaceutical compounds by determining the most favorable conformation of the ligand upon its interaction with the binding site of the desired protein [72, 73]. The investigated substances were subjected to molecular docking with the main protease (6LU7) protein in order to assess their efficacy as antiviral substances [38, 49, 71]. Molecular docking investigations facilitate the anticipation of binding characteristics through the utilization of simulated screening techniques and scoring features [74, 75]. This methodology explores the conformational compatibility between two molecular entities, namely a substance and the binding region of the desired receptor, akin to the intricate interlocking of puzzle pieces within a three-dimensional space.

The docking procedure was experimentally validated through the utilization of the re-docking and overlaying technique. The indigenous ligand derived from the 6LU7 structure was isolated and subsequently repositioned within the active region. The re-docking process was executed in order to assess the efficacy and efficiency of the docking methodology [76]. The identical methodology previously employed was utilized in the subsequent re-docking procedure. The re-docked complex was successfully aligned with the native ligand from 6LU7, resulting in a RMSD value of 1.014 Å (see Figure S40).

**Fig. 6:**
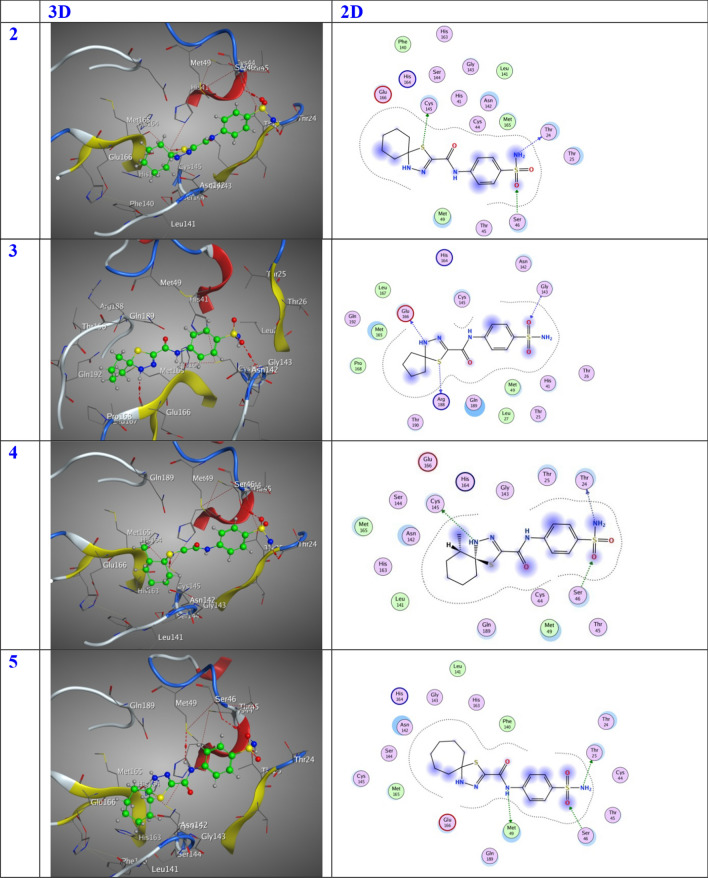

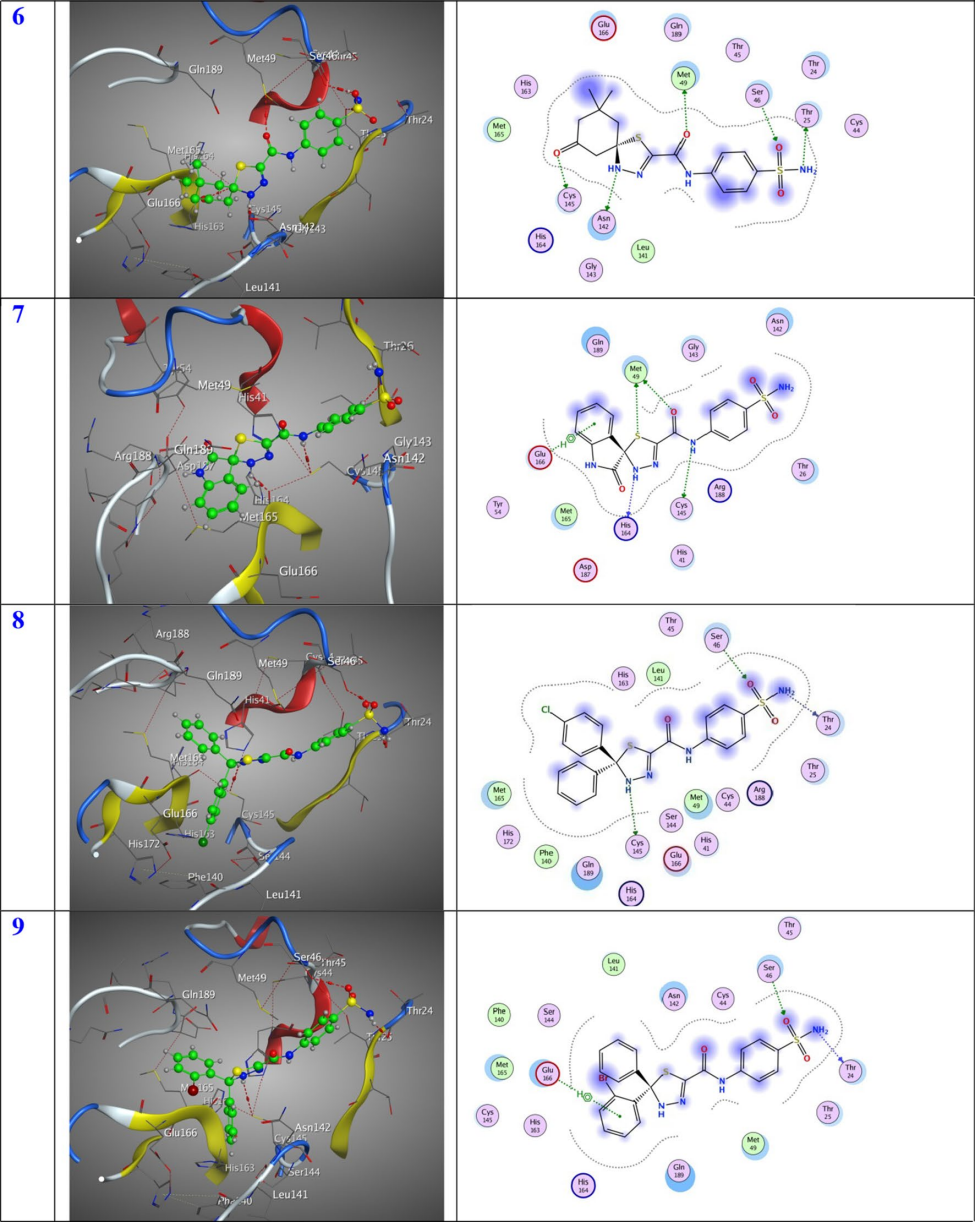

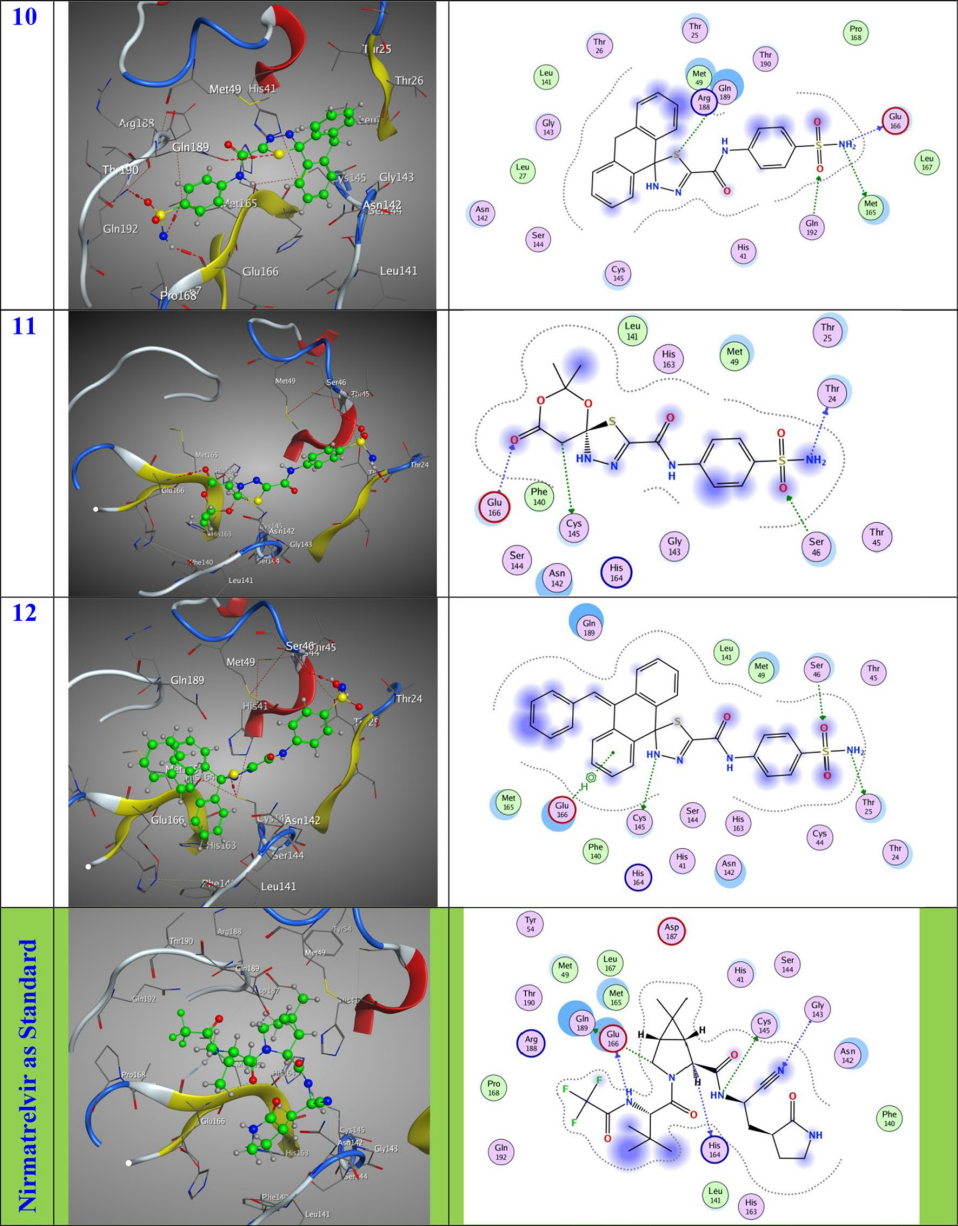
3D and 2D position of the title compounds inside the active site of 6lu7 protein

In this specific instance, the 6LU7 protein serves as the designated receptor, whereas the substances are regarded as substrates. The tabulated data in Table 1 present the outcomes of molecular docking, while Fig. 6 exhibits the most favorable arrangement of the substrates within the binding pocket. The substrates exhibit noteworthy favorable docking scores (S, kcal/mol), as illustrated in Table 1. The 6LU7 pocket is subject to diverse modes of interaction, including the establishment of hydrogen bonds and hydrophobic interactions. The observed phenomenon suggests a robust interplay between the docked substrates and the binding site of the receptor. The inhibitory activity degrees were ordered in the following manner: Compound **6** exhibits the lowest entropy (S) value of − 7.33 kcal/mol, followed by Compound **5** with a S value of − 7.22 kcal/mol. Compound **12** possesses a slightly higher S value of − 7.11 kcal/mol, while Compound **11** has a S value of − 6.88 kcal/mol. Compound **10** exhibits a lower S value of − 6.81 kcal/mol, followed by another Compound **10** with a S value of − 6.66 kcal/mol. Compound **4** and Compound **7** both have an identical S value of − 6.65 kcal/mol. Compound **3** possesses a slightly higher S value of − 6.61 kcal/mol, while Compound **9** and Compound **8** both have an identical S value of − 6.54 kcal/mol. It is intriguing to note that the compounds with the highest efficacy in the docking process are numbered 6, 5, 12, and 11, as indicated in Table 1. Compound **6** was effectively stabilized within the binding region through the establishment of specific intermolecular interactions. The docking score (S) of − 7.33 kcal/mol indicates a favorable binding affinity. These interactions involve the formation of three hydrogen-donor interactions with O10-MET49, N13-THR25, and N17-ASN142, as well as one hydrogen-acceptor interaction with O24-CYS145. In addition, an interaction between O14-SER46 is also observed. The distances between the interacting atoms are measured at 3.07, 2.95, 2.84, 3.43, and 2.87 Å, respectively. Additionally, compound **5** exhibited stabilization within the binding pocket through a docking score (S) of − 7.22 kcal/mol, facilitated by the establishment of two hydrogen-donor interactions with N7-MET49 and N13-THR25, as well as one hydrogen-acceptor interaction with O14-SER46. These interactions occurred at distances of 3.21, 2.94, and 3.02 Å, respectively. Compound **12** exhibited stabilization within the binding pocket through the establishment of various intermolecular interactions. The docking score (S) of − 7.11 kcal/mol signifies the favorable energy associated with this binding event. Specifically, two hydrogen-donor interactions were formed between N13-THR25, one hydrogen-acceptor interaction between N17-CYS145, and one pi-H interaction between O14-SER46 and the 6-ring-GLU166. These interactions occurred at distances of 2.96, 3.52, 2.85, and 4.42 Å, respectively. In contrast, compound **11** exhibited stabilization within the binding pocket, as evidenced by a docking score (S of − 6.88 kcal/mol. This stabilization was achieved through the formation of two hydrogen-donor and two hydrogen-acceptor interactions involving N13- THR24, C22- CYS145, O23- GLU166, and O26-SER46, with distances of 3.12, 4.16, 3.11, and 2.86 Å, respectively.

**Table 1:** Molecular docking data

	Ligand	Receptor	Interaction	Distance	E (kcal/mol)	S (kcal/mol)	%
**2**	S 11	CYS 145	H-donor	3.30	− 1.40	− 6.81	79.74
	N 13	THR 24	H-donor	3.28	− 1.30		
	O 15	SER 46	H-acceptor	2.82	− 2.00		
**3**	S 11	ARG 188	H-donor	3.30	− 0.10	− 6.61	77.40
	N 17	GLU 166	H-donor	2.93	− 1.40		
	O 15	GLY 143	H-acceptor	3.03	− 1.70		
**4**	N 13	THR 24	H-donor	3.21	− 1.50	− 6.65	77.87
	N 17	CYS 145	H-donor	3.99	− 1.90		
	O 15	SER 46	H-acceptor	2.90	− 1.80		
**5**	N 7	MET 49	H-donor	3.21	− 0.90	− 7.22	84.54
	N 13	THR 25	H-donor	2.94	− 0.90		
	O 14	SER 46	H-acceptor	3.02	− 1.10		
**6**	O 10	MET 49	H-donor	3.07	− 0.70	− 7.33	85.83
	N 13	THR 25	H-donor	2.95	− 0.90		
	N 17	ASN 142	H-donor	2.84	− 1.60		
	O 24	CYS 145	H-donor	3.43	− 0.80		
	O 14	SER 46	H-acceptor	2.87	− 2.00		
**7**	N 7	CYS 145	H-donor	4.34	− 1.80	− 6.65	77.87
	O 10	MET 49	H-donor	3.40	-0.10		
	S 11	MET 49	H-donor	3.41	0.20		
	N 17	HIS 164	H-donor	2.80	-2.70		
	6-ring	GLU 166	pi-H	4.60	-1.00		
**8**	N 13	THR 24	H-donor	3.19	-1.60	-6.54	76.58
	N 17	CYS 145	H-donor	4.35	-1.30		
	O 15	SER 46	H-acceptor	2.91	-1.20		
**9**	N 13	THR 24	H-donor	3.24	-1.40	-6.54	76.58
	O 15	SER 46	H-acceptor	2.92	-1.30		
	6-ring	GLU 166	pi-H	4.41	-1.70		
**10**	S 11	GLN 189	H-donor	3.77	-1.30	-6.66	77.99
	N 13	MET 165	H-donor	4.17	-1.90		
	N 13	GLU 166	H-donor	2.93	-3.10		
	O 15	GLN 192	H-acceptor	2.98	-1.00		
**11**	N 13	THR 24	H-donor	3.12	-1.70	-6.88	80.56
	C 22	CYS 145	H-donor	4.16	-0.60		
	O 23	GLU 166	H-acceptor	3.11	-1.30		
	O 26	SER 46	H-acceptor	2.86	-1.50		
**12**	N 13	THR 25	H-donor	2.96	-1.30	-7.11	83.26
	N 17	CYS 145	H-donor	3.52	-1.50		
	O 14	SER 46	H-acceptor	2.85	-1.50		
	6-ring	GLU 166	pi-H	4.42	-0.60		
**Nirmatrelvir**	N9	CYS 145	H-donor	3.14	-3.20	-8.54	
	N10	GLU 166	H-donor	2.87	-5.40		
	C16	HIS 164	H-donor	3.24	-1.30		
	C17	GLN 189	H-donor	3.16	-0.80		
	N12	GLY 143	H-acceptor	3.60	-0.60

The Nirmatrelvir as standard drug was used as standard for docking studies and for comparison of docking scores with the investigated compounds. The docking score (S) of the tested compounds was compared with that of the Nirmatrelvir as standard drug and the activity percent (% = (S_test_ / S_standard_)/100) was calculated, Table 1. The activity percent (%) of the current investigated compounds ranged from highest activity percent of 85.83% (in the case of compound **6**) and 84.54% (in the case of compound **5**) to the lowest activity percent of 76.58% (in the case of compounds **8** and **9**), Table 1.

### Density functional theory (DFT) analysis

**Fig. 7:**
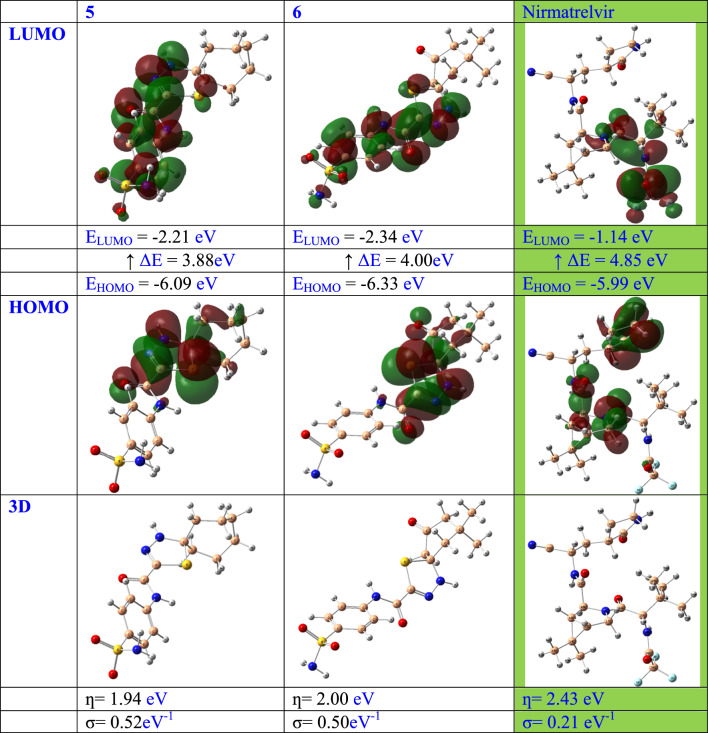
DFT exploration of compounds (**5** and **6**) and reference drug Nirmatrelvir

The DFT analysis was conducted using the B3LYP functional and a 6-31g + (d,p) basis set. Figure 7 displays the DFT assessments of the two highest-ranking compounds (**5** and **6**) obtained from the screening method, as well as the reference drug Nirmatrelvir. The results of the DFT analysis demonstrated that the HOMO–LUMO energy difference (ΔE) of compounds **5** and **6** (3.88 eV, and 4.00 eV, respectively) was lower compared to that of Nirmatrelvir (4.85eV). This finding indicates the possibility and significance of molecular charge transfer [77–79].

Furthermore, the hardness (ƞ) and softness (σ) values of the two highest-ranking compounds (**5** and **6**) identified by the screening approach, together with the reference drug Nirmatrelvir, were determined by evaluating the energies of their HOMOs and LUMOs orbitals using the Parr and Pearson interpretation [80–83]. Interestingly, compounds (**5** and **6**) had the highest chemical softness (0.52 eV^−1^, and 0.50 eV^−1^, respectively) and lowest chemical hardness (1.94 eV, and 2.00 eV, respectively) compared to that of Nirmatrelvir (0.21eV^−1^ and 2.43 eV, respectively) values which may contribute the higher chemical reactivity compared to Nirmatrelvir.

## Conclusion

New series of 1,3,4-thiadiazole derivatives have been synthesized from 2-hydrazinyl-N-(4-sulfamoylphenyl)-2-thioxoacetamide using a well-established method that has several advantages to afford a novel spiro heterocyclic compounds based on 1,3,4-thiadiazole derivatives hybrid with sulfamoylphenyl as a two bioisosteres moieties. We have confirmed the structure of these compounds by using spectral analysis techniques. Also, the drug-likeness properties of these compounds, such as Lipinski’s rule and ADMET (absorption, distribution, metabolism, excretion, and toxicity) profiles were evaluated using the ADMETlab 2.0 tool. Ten FDA-approved drugs for COVID-19 treatment were selected as reference pharmacophores to identify the structural features required for COVID-19 inhibition. Molecular docking against the 6LU7 protein had been performed to investigate the antiviral potential of these compounds and to analyze their binding interactions. The docking results showed that these compounds had good binding affinity to the COVID-19 main protease, with binding energy scores ranging from − 7.33 kcal/mol (compound **6**) and − 7.22kcal/mol (compound **5**) to − 6.54 kcal/mol (compounds **8** and **9**). Furthermore, density functional theory (DFT) analysis was performed on the two most promising compounds (**5** and **6**) that have been identified through the screening methodology, along with the reference drug Nirmatrelvir. The DFT analysis showed that compounds **5** and **6** had a lower HOMO–LUMO energy gap compared to Nirmatrelvir. This finding explains the potential and significance of intermolecular charge transfer. Here, this study greatly expands the chemical diversity of SARS-CoV-2 Mpro inhibitors and provides new building blocks for coronavirus antiviral drug discovery. Further studies are needed to validate the antiviral activity of these compounds in vivo.

### Supplementary Information

Below is the link to the electronic supplementary material.

Supplementary file1 (DOCX 10205 kb)

## References

[1] Sacks D, Ledwaba J, Morris L, Hunt GM (2017). Rapid detection of common HIV-1 drug resistance mutations by use of high-resolution melting analysis and unlabeled probes. J Clin Microbiol.

[2] Dhama K, Khan S, Tiwari R (2020). Coronavirus disease 2019–COVID-19. Clin Microbiol Rev.

[3] Liu C, Zhou Q, Li Y (2020). Research and development on therapeutic agents and vaccines for COVID-19 and related human coronavirus diseases. ACS Cent Sci.

[4] Balaramnavar VM, Ahmad K, Saeed M (2020). Pharmacophore-based approaches in the rational repurposing technique for FDA approved drugs targeting SARS-CoV-2 Mpro. RSC Adv.

[5] Li Y, Geng J, Liu Y (2013). Thiadiazole-a promising structure in medicinal chemistry. ChemMedChem.

[6] Vernazza P, Wang C, Pozniak A (2013). Efficacy and safety of Lersivirine (UK-453,061) versus Efavirenz in antiretroviral treatment-naive HIV-1–infected patients: week 48 primary analysis results from an ongoing, multicenter, randomized, double-blind, Phase IIb trial. JAIDS J Acquired Immune Defic Syndromes.

[7] Hu Y, Li CY, Wang XM (2014). 1,3,4-Thiadiazole: Synthesis, reactions, and applications in medicinal, agricultural, and materials chemistry. Chem Rev.

[8] Jain AK, Sharma S, Vaidya A (2013). 1,3,4-thiadiazole and its derivatives: a review on recent progress in biological activities. Chem Biol Drug Des.

[9] Anthwal T, Singh HO, Nain S (2022). 1,3,4-Thiadiazole scaffold: anti-microbial agents. Pharm Chem J.

[10] Janowska S, Paneth A, Wujec M (2020). Cytotoxic properties of 1,3,4-Thiadiazole derivatives—a review. Molecules.

[11] El Fal M, Ramli Y, Zerzouf A (2015). Synthesis and antibacterial activity of new spiro[thiadiazoline-(pyrazolo[3,4-d]pyrimidine)] derivatives. J Chem.

[12] Anthwal T, Nain S (2022). 1,3,4-Thiadiazole scaffold: as anti-epileptic agents. Front Chem.

[13] Aliabadi A (2016). 1,3,4-Thiadiazole based anticancer agents. Anticancer Agents Med Chem.

[14] Ergena A, Rajeshwar Y, Solomon G (2022). Synthesis and diuretic activity of substituted 1,3,4-thiadiazoles. Scientifica.

[15] De Lourdes G, Ferreira M, Pinheiro LCS, Santos-Filho OA (2014). Design, synthesis, and antiviral activity of new 1H–1,2,3-triazole nucleoside ribavirin analogs. Med Chem Res.

[16] Banerjee R, Perera L, Tillekeratne LMV (2021). Potential SARS-CoV-2 main protease inhibitors. Drug Discov Today.

[17] Kumar D, Kumar H, Kumar V (2023). Mechanism-based approaches of 1,3,4 thiadiazole scaffolds as potent enzyme inhibitors for cytotoxicity and antiviral activity. Med Drug Discov.

[18] Anthwal T, Paliwal S, Nain S (2022). Diverse Biological Activities of 1,3,4-Thiadiazole Scaffold. Chemistry (Switzerland).

[19] Mercorelli B, Desantis J, Celegato M (2022). Discovery of novel SARS-CoV-2 inhibitors targeting the main protease Mpro by virtual screenings and hit optimization. Antiviral Res.

[20] Rabie AM, Eltayb WA (2023). Potent dual polymerase/exonuclease inhibitory activities of antioxidant aminothiadiazoles against the COVID-19 Omicron Virus: a promising in silico/in vitro repositioning research study. Mol Biotechnol.

[21] Rashdan HRM, Abdelmonsef AH (2022). In silico study to identify novel potential thiadiazole-based molecules as anti-Covid-19 candidates by hierarchical virtual screening and molecular dynamics simulations. Struct Chem.

[22] Rashdan HRM, Abdelmonsef AH (2022). Towards Covid-19 TMPRSS2 enzyme inhibitors and antimicrobial agents: synthesis, antimicrobial potency, molecular docking, and drug-likeness prediction of thiadiazole-triazole hybrids. J Mol Struct.

[23] Elkanzi NAA, Kadry AM, Ryad RM (2022). Efficient and recoverable bio-organic catalyst cysteine for synthesis, docking study, and antifungal activity of new bio-active 3,4-dihydropyrimidin-2(1 H)-ones/thiones under microwave irradiation. ACS Omega.

[24] Mohamed MAA, Bekhit AA, Allah OAA (2021). Synthesis and antimicrobial activity of some novel 1,2-dihydro-[1,2,4]triazolo[1,5-: a] pyrimidines bearing amino acid moiety. RSC Adv.

[25] El-Saghier AM, Abosella L, Aborahma GA (2023). Synthesis and insecticide evaluation of some new oxopropylthiourea compounds as insect growth regulators against the cotton leafworm, *Spodoptera littoralis*. Sci Rep.

[26] El-Saghier AM, Abd El-Halim HF, Abdel-Rahman LH, Kadry A (2019). Green synthesis of new trizole based heterocyclic amino acids ligands and their transition metal complexes. Characterization, kinetics, antimicrobial and docking studies. Appl Organomet Chem.

[27] Abd Allah OA, El-Saghier AM, Kadry AM (2015). Synthesis, structural stability calculation, and antibacterial evaluation of novel 3,5-diphenylcyclohex-2-en-1-one derivatives. Synth Commun.

[28] Abd Allah OA, El-Saghier AM, Kadry AM, Seleem AA (2015). Synthesis and evaluation of some novel curcumin derivatives as anti-inflammatory agents. Int J Pharm Sci Rev Res.

[29] Abdelmonsef AH, El-Saghier AM, Kadry AM (2023). Ultrasound-assisted green synthesis of triazole-based azomethine/thiazolidin-4-one hybrid inhibitors for cancer therapy through targeting dysregulation signatures of some Rab proteins. Green Chem Lett Rev.

[30] El-Saghier AMM, Mohamed MAA, Abdalla OA, Kadry AM (2018). Utility of amino acid coupled 1,2,4-triazoles in organic synthesis: synthesis of some new antileishmainal agents. Bull Chem Soc Ethiop.

[31] Mohamed MAA, Abd Allah OA, Bekhit AA (2020). Synthesis and antidiabetic activity of novel triazole derivatives containing amino acids. J Heterocycl Chem.

[32] El-Saghier AM, Mohamed MA, Abd-Allah OA (2019). Green synthesis, antileishmanial activity evaluation, and in silico studies of new amino acid-coupled 1,2,4-triazoles. Med Chem Res.

[33] El-Saghier AM, Abdou A, Mohamed MAA (2023). Novel 2-acetamido-2-ylidene-4-imidazole derivatives (El-Saghier reaction): green synthesis, biological assessment, and molecular docking. ACS Omega.

[34] El-Saghier AM, Abdul-Baset A, El-Hady OM, Kadry AM (2023). Synthesis of some new thiadiazole/thiadiazine derivatives as potent biologically active compounds. Sohag J Sci.

[35] Yarovenko VN, Shirokov AV, Krupinova ON (2003). Synthesis of oxamic acids thiohydrazides and carbamoyl-1,3,4-thiadiazoles. Russ J Org Chem.

[36] Aksenov AN, Krayushkin MM, Yarovenko VN (2021). Synthesis of (2-chloroquinolin-3-yl)-1,3,4-thiadiazole-2-carboxamides. Russ Chem Bull.

[37] Xiong G, Wu Z, Yi J (2021). ADMETlab 2.0: an integrated online platform for accurate and comprehensive predictions of ADMET properties. Nucleic Acids Res.

[38] Shaaban S, Abdou A, Alhamzani AG (2023). Synthesis and in silico investigation of organoselenium-clubbed schiff bases as potential Mpro inhibitors for the SARS-CoV-2 replication. Life.

[39] Mishra GP, Sharma R (2016). Identification of potential PPAR γ agonists as hypoglycemic agents: molecular docking approach. Interdisciplinary Sci.

[40] Mishra GP, Bhadane RN, Panigrahi D (2021). The interaction of the bioflavonoids with five SARS-CoV-2 proteins targets: an in silico study. Comput Biol Med.

[41] Scholz C, Knorr S, Hamacher K, Schmidt B (2015). DOCKTITE-A highly versatile step-by-step workflow for covalent docking and virtual screening in the molecular operating environment. J Chem Inf Model.

[42] Arafath MA, Adam F, Ahamed MBK (2023). Ni(II), Pd(II) and Pt(II) complexes with SNO-group thiosemicarbazone and DMSO: synthesis, characterization, DFT, molecular docking and cytotoxicity. J Mol Struct.

[43] Abd El-Lateef HM, Khalaf MM, Kandeel M (2023). New mixed-ligand thioether-quinoline complexes of nickel(II), cobalt(II), and copper(II): Synthesis, structural elucidation, density functional theory, antimicrobial activity, and molecular docking exploration. Appl Organomet Chem.

[44] Latif MA, Ahmed T, Hossain MS (2023). Synthesis, spectroscopic characterization, DFT calculations, antibacterial activity, and molecular docking analysis of Ni(II), Zn(II), Sb(III), and U(VI) metal complexes derived from a nitrogen-sulfur schiff base. Russ J Gen Chem.

[45] Abd El-Lateef HM, Khalaf MM, Kandeel M (2023). Designing, characterization, biological, DFT, and molecular docking analysis for new FeAZD, NiAZD, and CuAZD complexes incorporating 1-(2-hydroxyphenylazo)−2-naphthol (H2AZD). Comput Biol Chem.

[46] Jin Z, Du X, Xu Y (2020). Structure of Mpro from SARS-CoV-2 and discovery of its inhibitors. Nature.

[47] Abd El-Lateef HM, Khalaf MM, Kandeel M, Abdou A (2023). Synthesis, characterization, DFT, biological and molecular docking of mixed ligand complexes of Ni(II), Co(II), and Cu(II) based on ciprofloxacin and 2-(1H-benzimidazol-2-yl)phenol. Inorg Chem Commun.

[48] Abd El-Lateef HM, Khalaf MM, Amer AA (2023). Synthesis, characterization, antimicrobial, density functional theory, and molecular docking studies of novel Mn(II), Fe(III), and Cr(III) complexes incorporating 4-(2-hydroxyphenyl azo)-1-naphthol (Az). ACS Omega.

[49] Shaaban S, Al-Faiyz YS, Alsulaim GM (2023). Synthesis of new organoselenium-based succinanilic and maleanilic derivatives and in silico studies as possible SARS-CoV-2 main protease inhibitors. Inorganics.

[50] Najar AM, Eswayah A, Moftah MB et al (2023) Rigidity and Flexibility of Pyrazole, s-Triazole, and v-Triazole Derivative of Chloroquine as Potential Therapeutic against COVID-19. J Med Chem Sci 6:2056–2084. 10.26655/JMCHEMSCI.2023.9.14

[51] Jereva D, Alov P, Tsakovska I (2022). Application of InterCriteria analysis to assess the performance of scoring functions in molecular docking software packages. Mathematics.

[52] Shivanika C, Deepak Kumar S, Ragunathan V (2022). Molecular docking, validation, dynamics simulations, and pharmacokinetic prediction of natural compounds against the SARS-CoV-2 main-protease. J Biomol Struct Dyn.

[53] Gleeson MP, Gleeson D (2009). QM/MM calculations in drug discovery: A useful method for studying binding phenomena?. J Chem Inf Model.

[54] Abdou A, Omran OA, Al-Fahemi JH (2023). Lower rim thiacalixarenes derivatives incorporating multiple coordinating carbonyl groups: synthesis, characterization, ion-responsive ability and DFT computational analysis. J Mol Struct.

[55] Becke AD (1988). Density-functional exchange-energy approximation with correct asymptotic behavior. Phys Rev A.

[56] Lee C, Yang W, Parr RG (1988). Development of the Colle-Salvetti correlation-energy formula into a functional of the electron density. Phys Rev B.

[57] Kruse H, Goerigk L, Grimme S (2012). Why the standard B3LYP/6-31G* model chemistry should not be used in DFT calculations of molecular thermochemistry: understanding and correcting the problem. J Org Chem.

[58] Tomberg A (2013) An Introduction to Computational Chemistry Using G09W and Avogadro Software. Gaussian 09W Tutorial

[59] Albayati MR, Kansız S, Dege N (2020). Synthesis, crystal structure, Hirshfeld surface analysis and DFT calculations of 2-[(2,3-dimethylphenyl)amino]-N’-[(E)-thiophen-2-ylmethylidene]benzohydrazide. J Mol Struct.

[60] Singh VK, Chaurasia H, Kumari P (2022). Design, synthesis, and molecular dynamics simulation studies of quinoline derivatives as protease inhibitors against SARS-CoV-2. J Biomol Struct Dyn.

[61] Hrichi H, Elkanzi NAA, Ali AM, Abdou A (2023). A novel colorimetric chemosensor based on 2-[(carbamothioylhydrazono) methyl]phenyl 4-methylbenzenesulfonate (CHMPMBS) for the detection of Cu(II) in aqueous medium. Res Chem Intermed.

[62] El-Shafei AK, El-Saghier AMM, Ahmed EA (1994). Synthesis of some new spiro(pyran-4,2’-benzoxazole) derivatives. Synthesis.

[63] Nayak YN, Gaonkar SL, Sabu M (2023). Chalcones: versatile intermediates in heterocyclic synthesis. J Heterocycl Chem.

[64] Mohamed MAA, Kadry AM, Farghaly MM, El-Saghier AMM (2021). Synthesis, characterization and antibacterial activity of some novel spiro[naphtho[1,2-e][1,3]oxazine-3,4’-pyran] derivatives. J Pharm Appl Chem.

[65] El-Saghier AM, Abd Allah OA, Kadry AM (2013). Design, synthesis and antibacterial evaluation of some new 3,5-diphenylcyclohex-2-en-1-one derivatives. J Adv Chem.

[66] Lipinski CA, Lombardo F, Dominy BW, Feeney PJ (2012). Experimental and computational approaches to estimate solubility and permeability in drug discovery and development settings. Adv Drug Deliv Rev.

[67] Owens J, Lipinski CA (2003). Chris Lipinski discusses life and chemistry after the Rule of Five. Drug Discov Today.

[68] Maji S, Pattanayak SK, Sen A, Badavath VN, Rudrapal M, Egbuna C (2022). Pharmacophore modeling in drug design. Computer aided drug design (CADD): from ligand-based methods to structure-based approaches.

[69] Sahu SN, Satpathy SS, Pattnaik S (2022). Boerhavia diffusa plant extract can be a new potent therapeutics against mutant nephrin protein responsible for type1 nephrotic syndrome: Insight into hydrate-ligand docking interactions and molecular dynamics simulation study. J Indian Chem Soc.

[70] Moharana M, Pattanayak SK, Khan F (2023) Molecular recognition of bio-active triterpenoids from Swertia chirayita towards hepatitis Delta antigen: a mechanism through docking, dynamics simulation, Gibbs free energy landscape. J Biomol Struct Dyn. 10.1080/07391102.2023.2184173 10.1080/07391102.2023.2184173 36856037

[71] Mengist HM, Fan X, Jin T (2020). Designing of improved drugs for COVID-19: Crystal structure of SARS-CoV-2 main protease Mpro. Signal Transduct Target Ther.

[72] Abd El-Lateef HM, Khalaf MM, El-Taib Heakal F, Abdou A (2023). Fe(III), Ni(II), and Cu(II)-moxifloxacin-tri-substituted imidazole mixed ligand complexes: synthesis, structural, DFT, biological, and protein-binding analysis. Inorg Chem Commun.

[73] El-Remaily MAEAAA, Elhady O, Abdou A (2023). Development of new 2-(Benzothiazol-2-ylimino)-2,3-dihydro-1H-imidazol-4-ol complexes as a robust catalysts for synthesis of thiazole 6-carbonitrile derivatives supported by DFT studies. J Mol Struct.

[74] Abu-Dief AM, El-Khatib RM, El-Dabea T (2023). Fabrication, structural elucidation of some new metal chelates based on N-(1H-Benzoimidazol-2-yl)-guanidine ligand: DNA interaction, pharmaceutical studies and molecular docking approach. J Mol Liq.

[75] Khalil EAM, Mahmoud WH, El Desssouky MMI, Mohamed GG (2021). Synthesis, spectral, thermal and biological studies of some transition and inner transition schiff base metal complexes. Egypt J Chem.

[76] Jarad AJ, Dahi MA, Al-Noor TH (2023). Synthesis, spectral studies, DFT, biological evaluation, molecular docking and dyeing performance of 1-(4-((2-amino-5-methoxy)diazenyl)phenyl) ethanone complexes with some metallic ions. J Mol Struct.

[77] Acar N, Selçuki C, Coşkun E (2017). DFT and TDDFT investigation of the Schiff base formed by tacrine and saccharin. J Mol Model.

[78] Bolognesi A, Porzio W, Provasoli A (2001). Structural and thermal behavior of poly (3-octylthiophene): a DSC, 13C MAS NMR, XRD, photoluminescence, and Raman scattering study. Macromol Chem Phys.

[79] Tandon H, Chakraborty T, Suhag V (2019). A brief review on importance of DFT in drug design. Res Med Eng Sci.

[80] Calais J-L (1993) Density-functional theory of atoms and molecules. In: Parr RG, Yang W (eds) Oxford University Press, New York, Oxford, 1989. IX + 333 pp. Price £45.00. In: International Journal of Quantum Chemistry. pp 101–101

[81] Pearson RG (1995). The HSAB Principle - more quantitative aspects. Inorg Chim Acta.

[82] Ben Hadda T, Berredjem M, Almalki FA (2022). How to face COVID-19: proposed treatments based on remdesivir and hydroxychloroquine in the presence of zinc sulfate. Docking/DFT/POM structural analysis. J Biomol Struct Dyn.

[83] Shokr EK, Kamel MS, Abdel-Ghany H (2022). Synthesis, characterization, and DFT study of linear and non-linear optical properties of some novel thieno[2,3-b]thiophene azo dye derivatives. Mater Chem Phys.

