# Supplementary material for: An efficient eco-friendly, simple, and green synthesis of some new spiro-N-(4-sulfamoyl-phenyl)-1,3,4-thiadiazole-2-carboxamide derivatives as potential inhibitors of SARS-CoV-2 proteases: drug-likeness, pharmacophore, molecular docking, and DFT exploration
Source: Mol Divers. 2023 Nov 9;28(1):249–70. doi: 10.1007/s11030-023-10761-0 (PMC10876818; doi:10.1007/s11030-023-10761-0)
Supplement: Supplementary file 1 — Supplementary file1 (DOCX 10205 kb) [file 11030_2023_10761_MOESM1_ESM.docx]

**An Efficient Eco-friendly, Simple and Green Synthesis of Some New Spiro-N-(4-sulfamoyl-phenyl)-1,3,4-thiadiazole-2-carboxamide Derivatives as Potential Inhibitors of SARS-CoV-2 Proteases: Drug-likeness, Pharmacophore, Molecular Docking and DFT Exploration**

Ahmed M. El-Saghier^*a^, Souhaila S. Enaili^a,b^, Aly Abdou^a^, Asmaa M. Kadry^a^

^a*^ Chemistry Department, Faculty of Science, Sohag University, Sohag 82524, Egypt.

[el.saghier@science.sohag.edu.eg](mailto:el.saghier@science.sohag.edu.eg).

^b^Chemistry Department, Faculty of Science, Al Zawiya University, Al Zawia, Libya.

**Spectral Analysis of the Newly Synthesized Compounds 1-12**


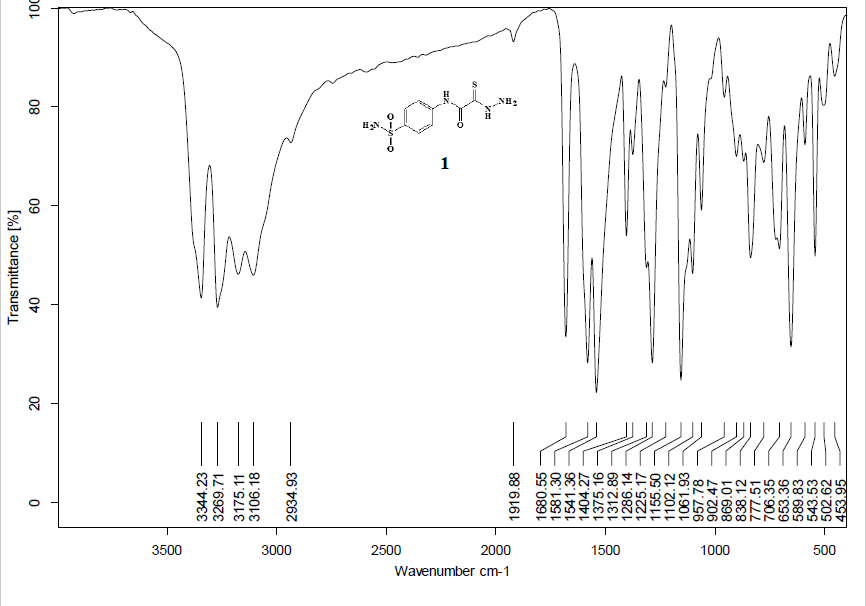


**Figure 1**: IR Spectrum of compound **1**


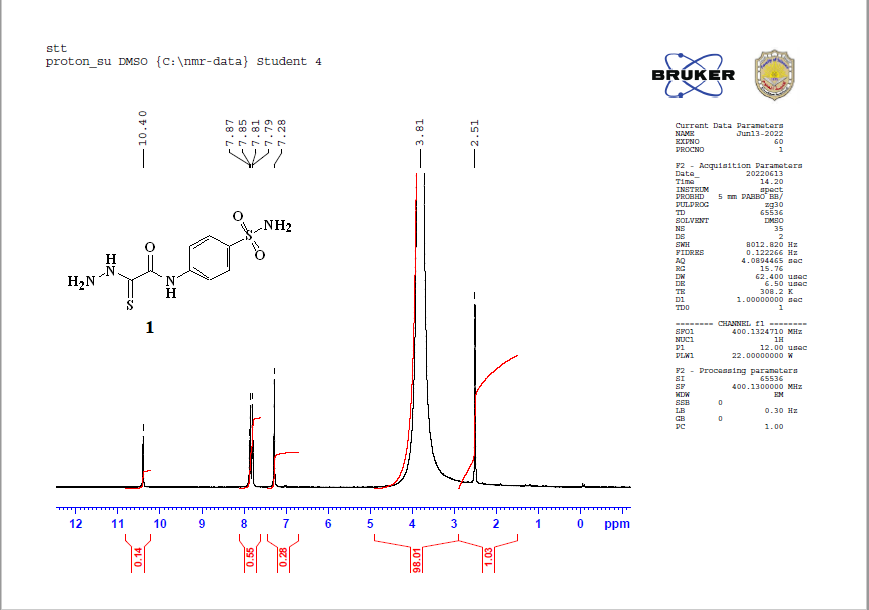


**Figure 2**: ^1^HNMR Spectrum of compound **1**


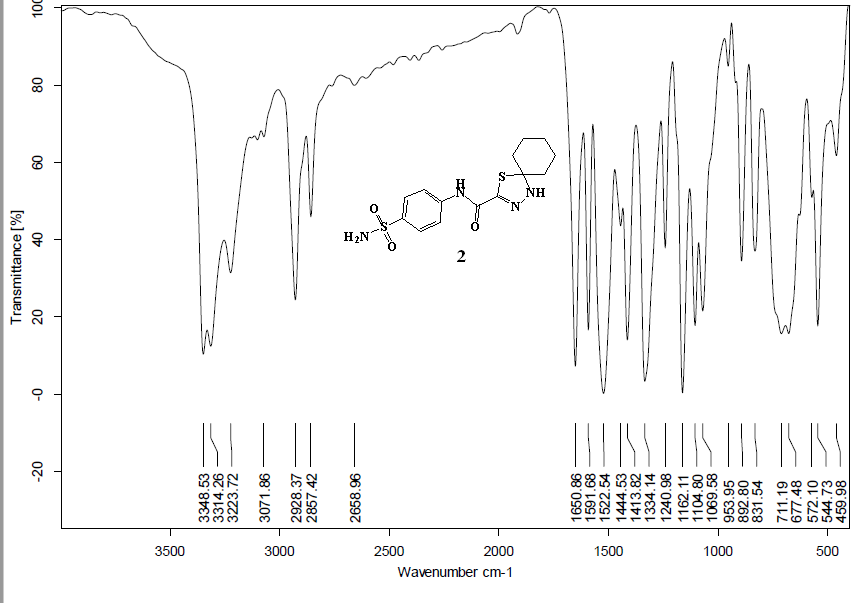


**Figure 3**: IR Spectrum of compound **2**


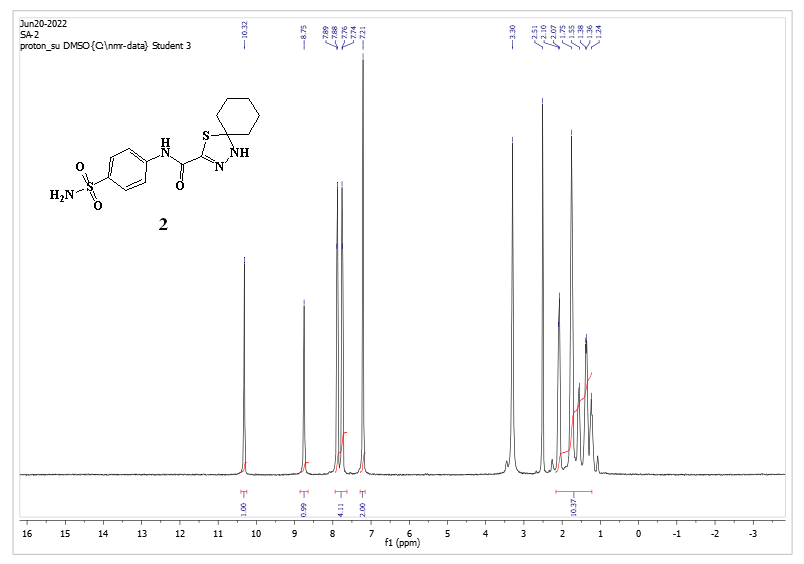


**Figure 4**: ^1^HNMR Spectrum of compound **2**


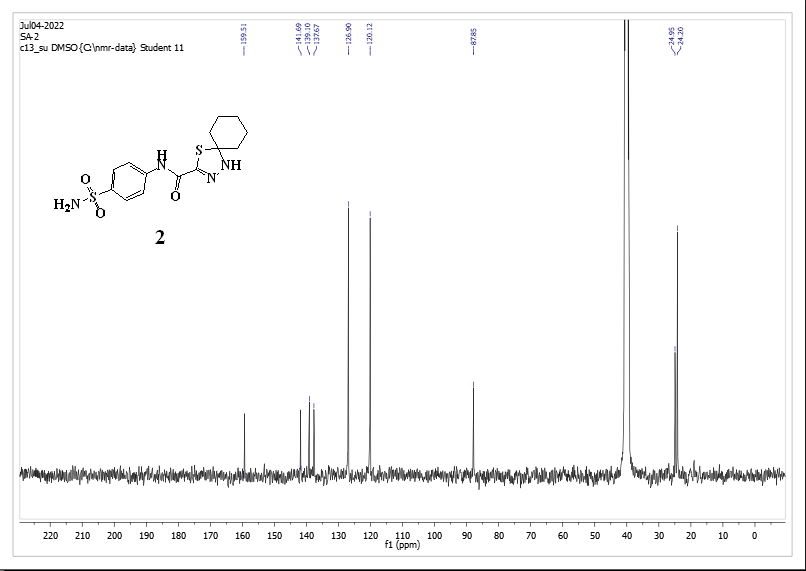


**Figure 5**: ^13^CNMR Spectrum of compound **2**


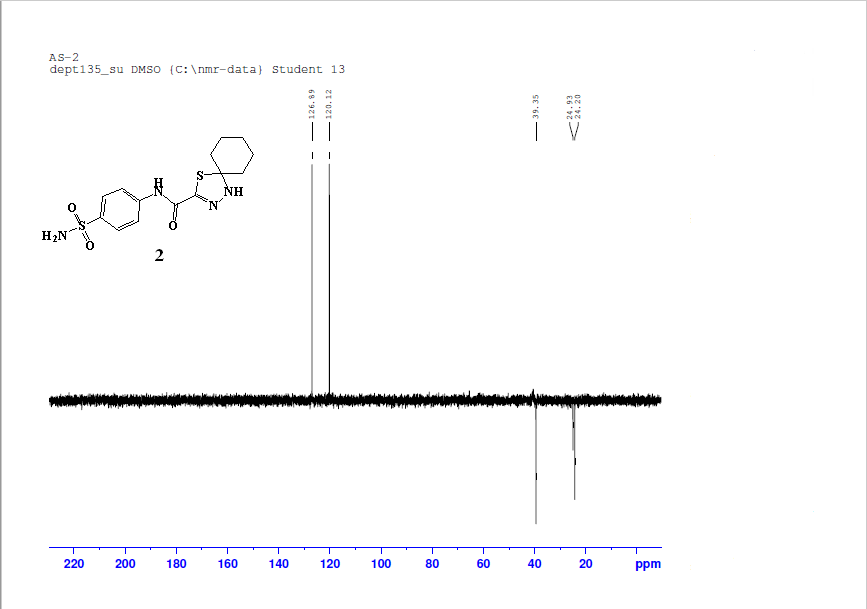


**Figure 6**: DEPT Spectrum of compound **2**


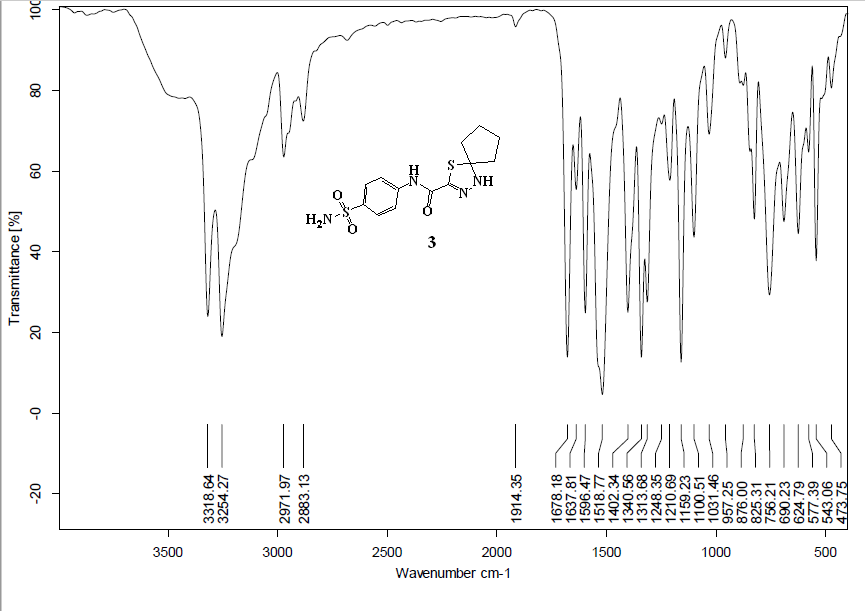


**Figure 7**: IR Spectrum of compound **3**


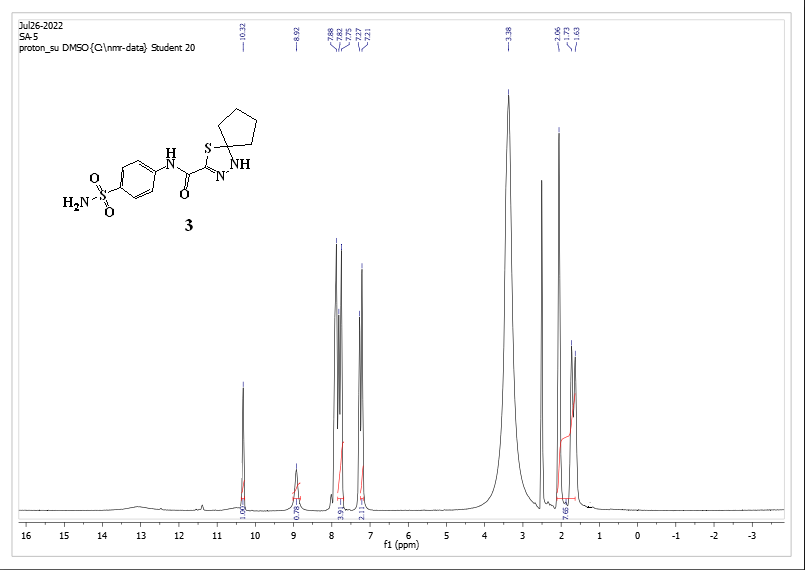


**Figure 8**: ^1^HNMR Spectrum of compound **3**


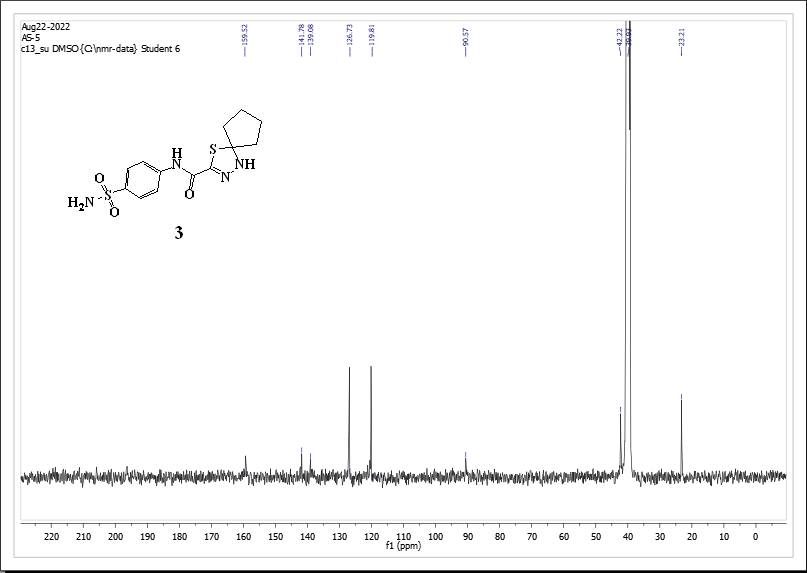


**Figure 9**: ^13^CNMR Spectrum of compound **3**


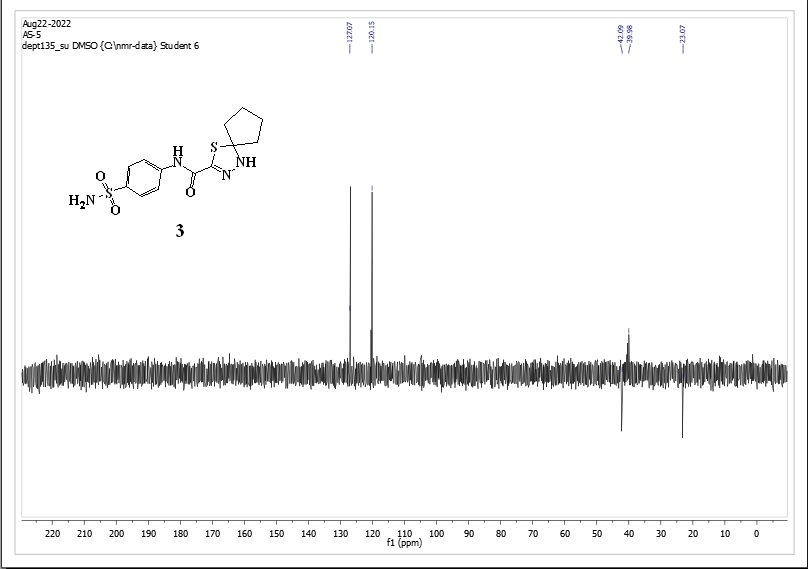


**Figure 10**: DEPT Spectrum of compound **3**


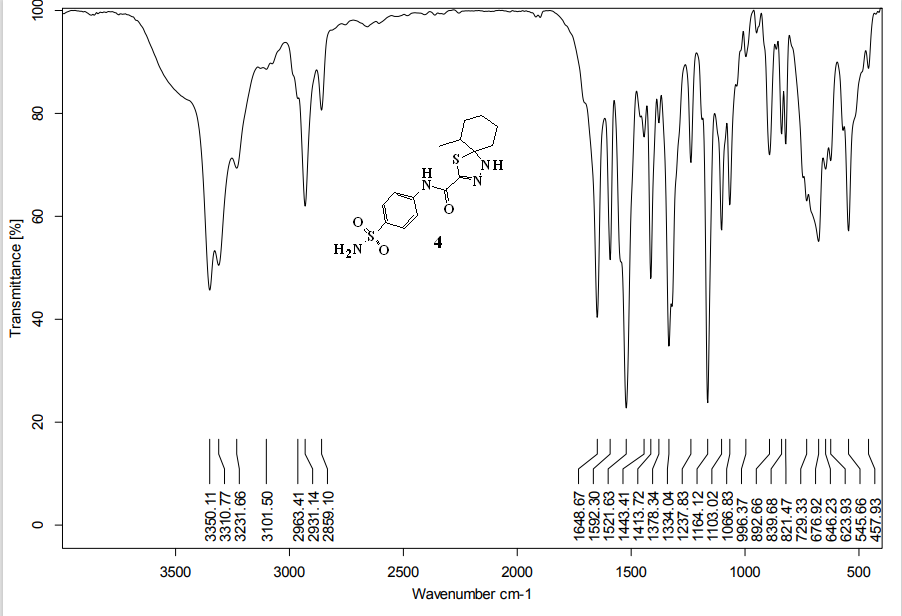


**Figure 11**: IR Spectrum of compound **4**


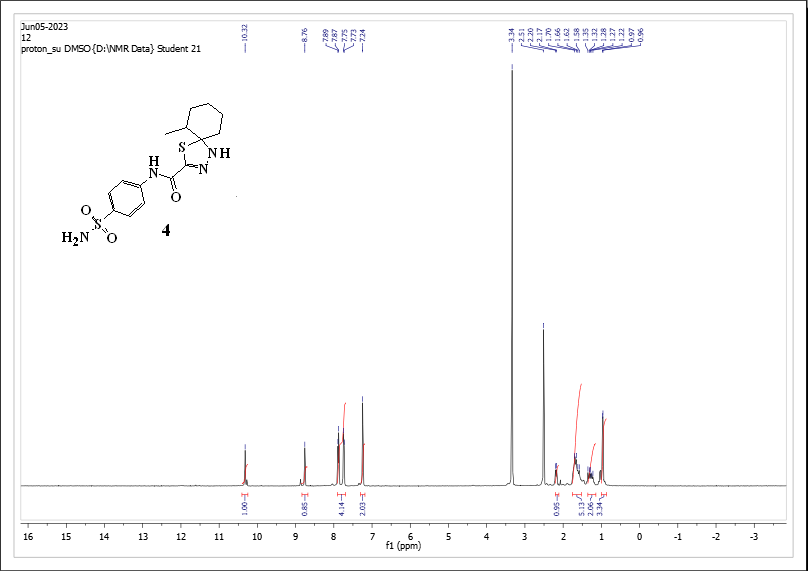


**Figure 11**: ^1^HNMR Spectrum of compound **4**


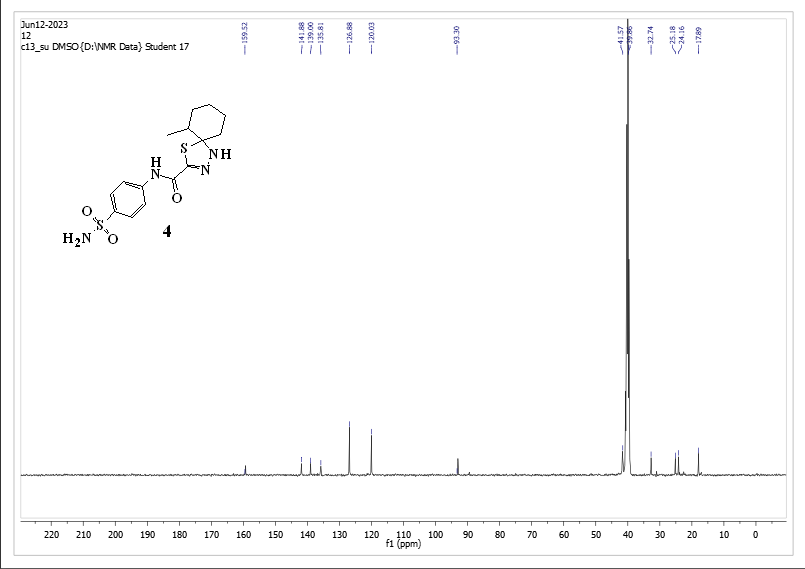


**Figure 12**: ^13^C-NMR Spectrum of compound **4**


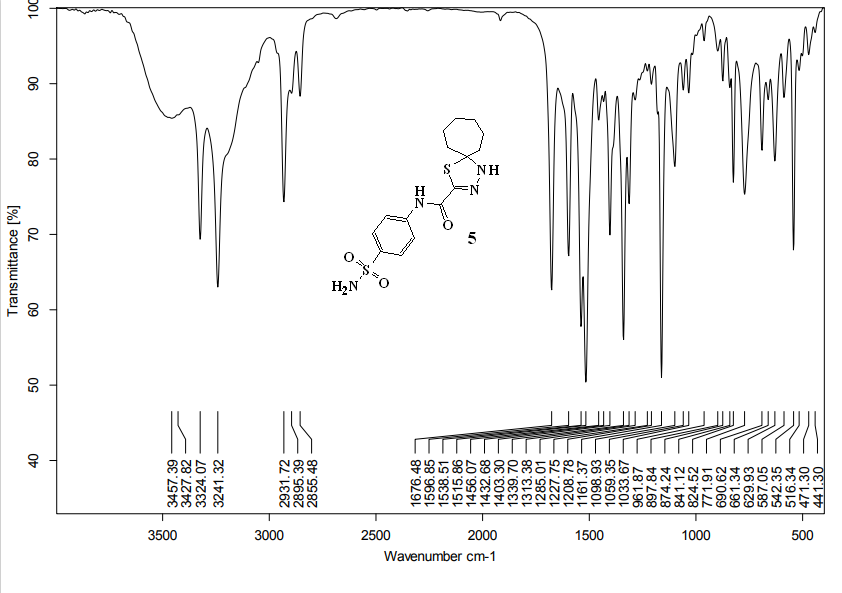


**Figure 13**: IR Spectrum of compound **5**


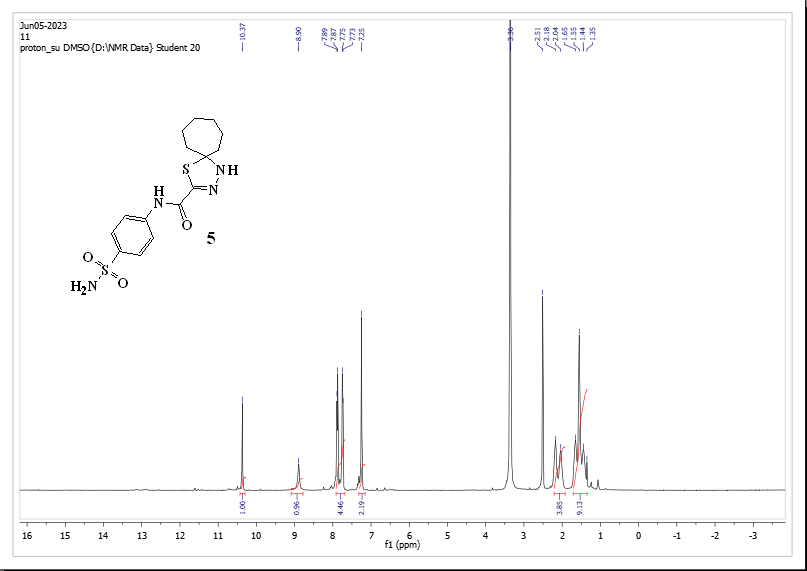


**Figure 14**: ^1^HNMR Spectrum of compound **5**


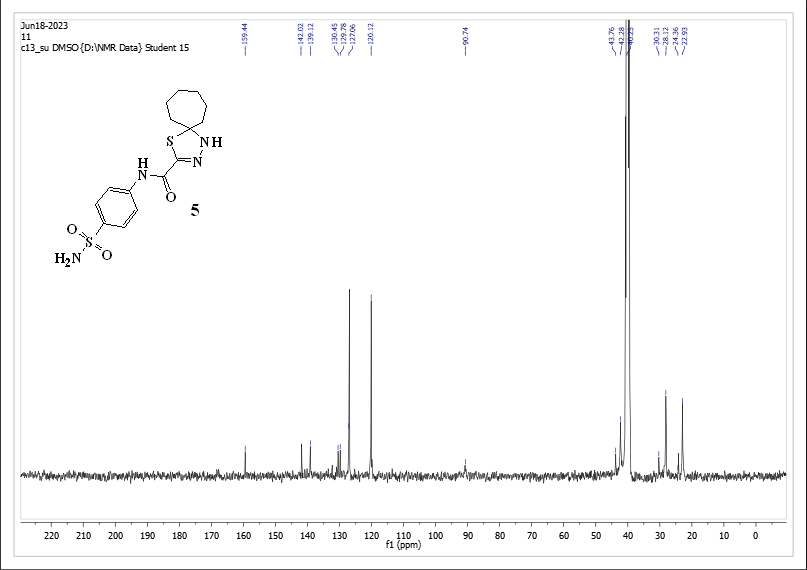


**Figure 15**: ^13^CNMR Spectrum of compound **5**


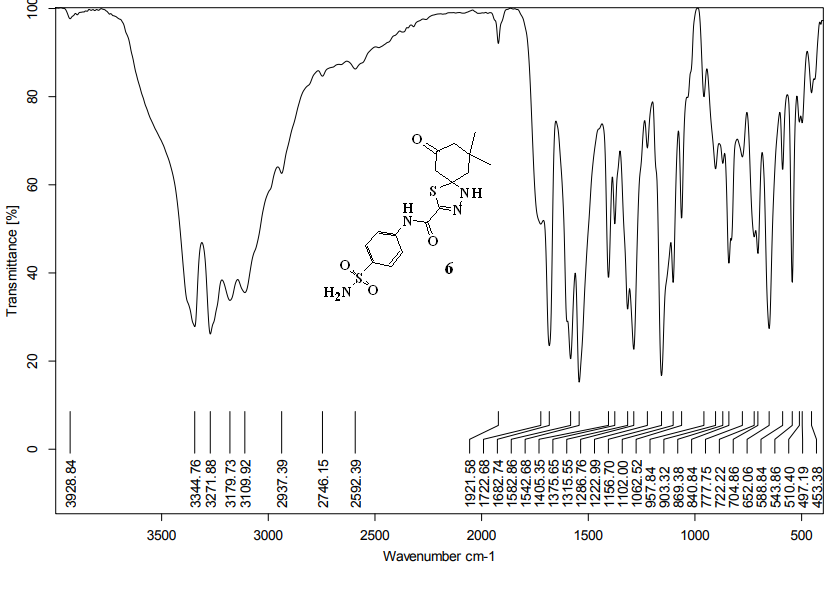


**Figure 16**: IR Spectrum of compound **6**


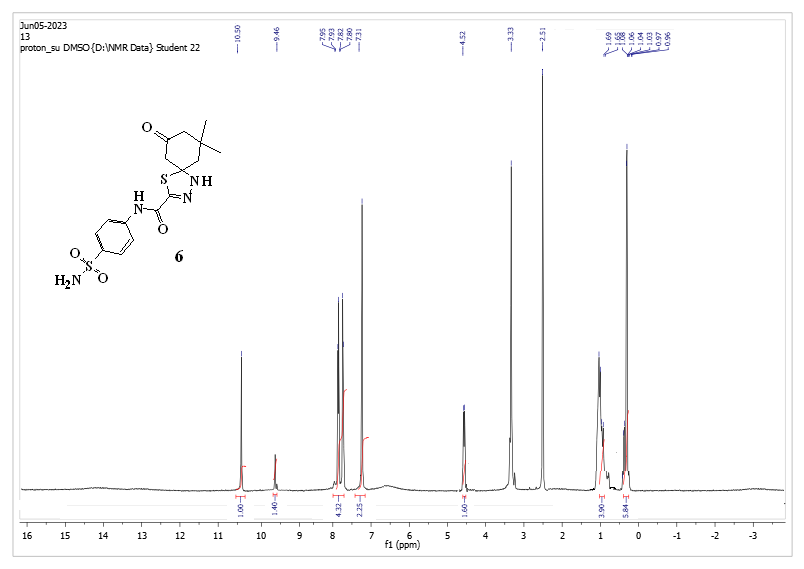


**Figure 17**: ^1^HNMR Spectrum of compound **6**


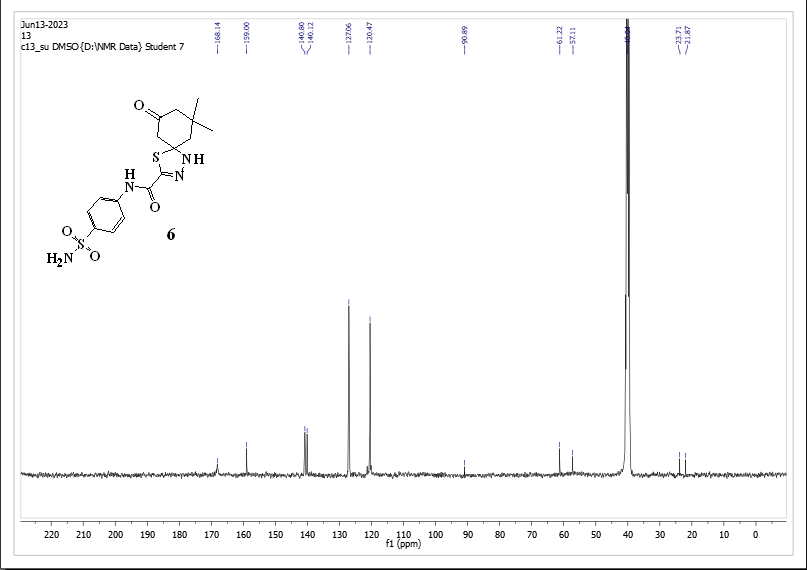


**Figure 18**: ^13^CNMR Spectrum of compound **6**


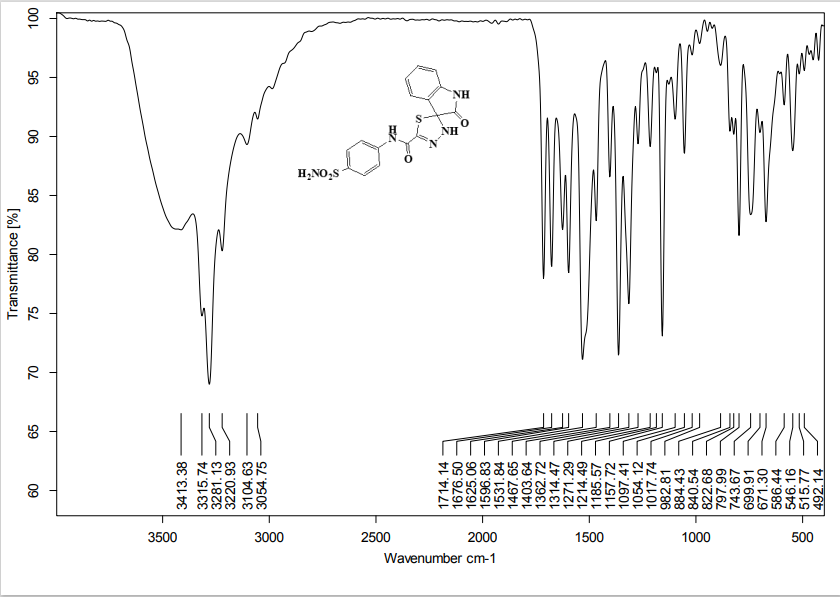


**Figure 19**: IR Spectrum of compound **7**


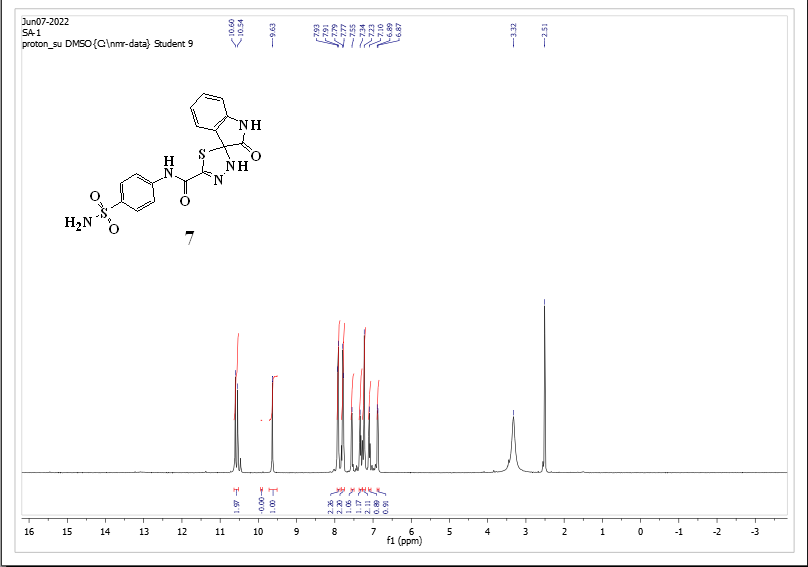


**Figure 20**: ^1^HNMR Spectrum of compound **7**


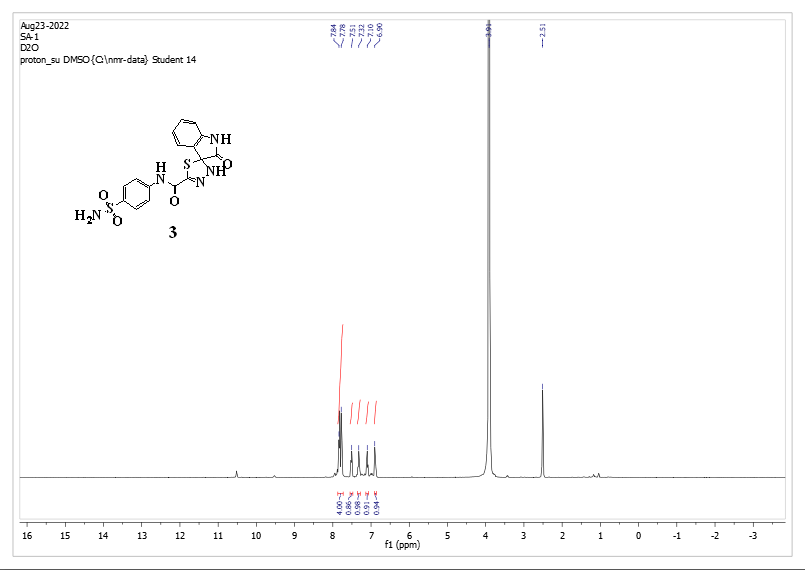


**Figure 21**: D_2_O Spectrum of compound **7**


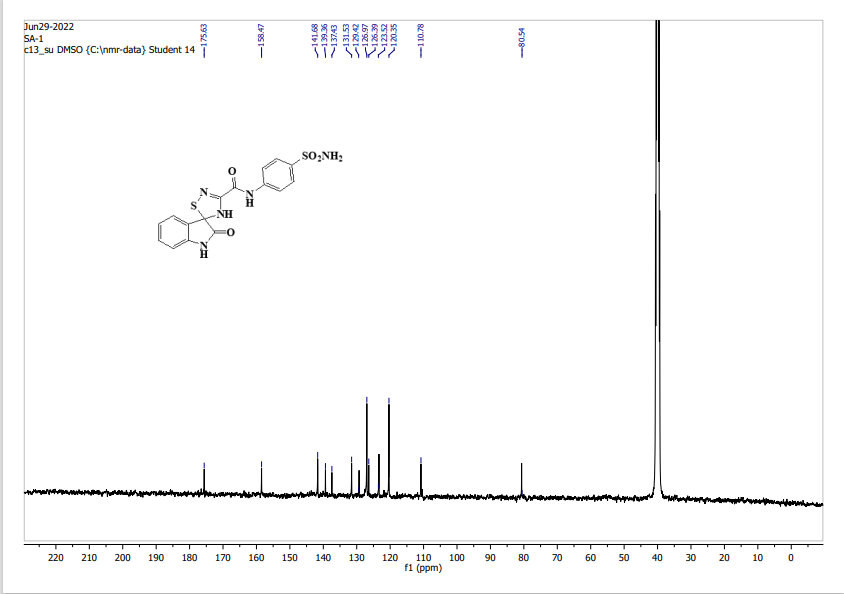


**Figure 22**: ^13^CNMR Spectrum of compound **7**


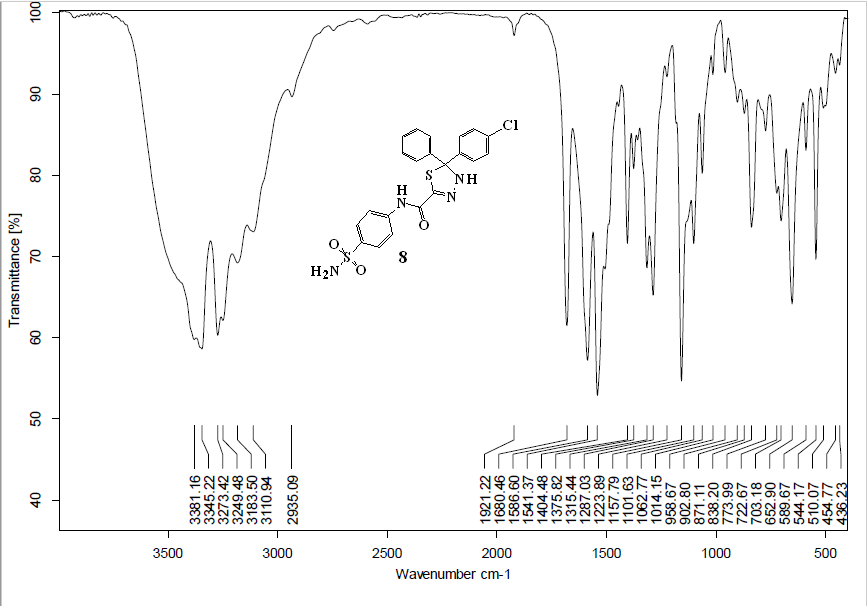


**Figure 23**: IR Spectrum of compound **8**


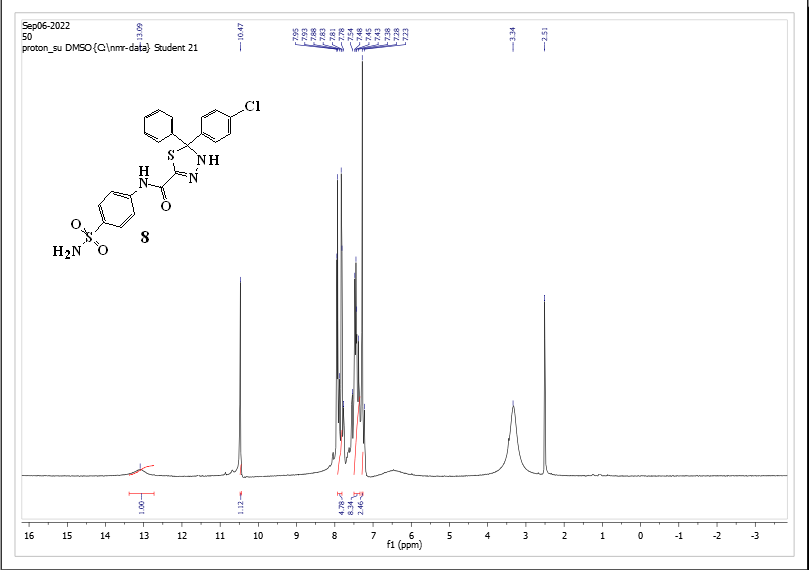


**Figure 24**: ^1^HNMR Spectrum of compound **8**


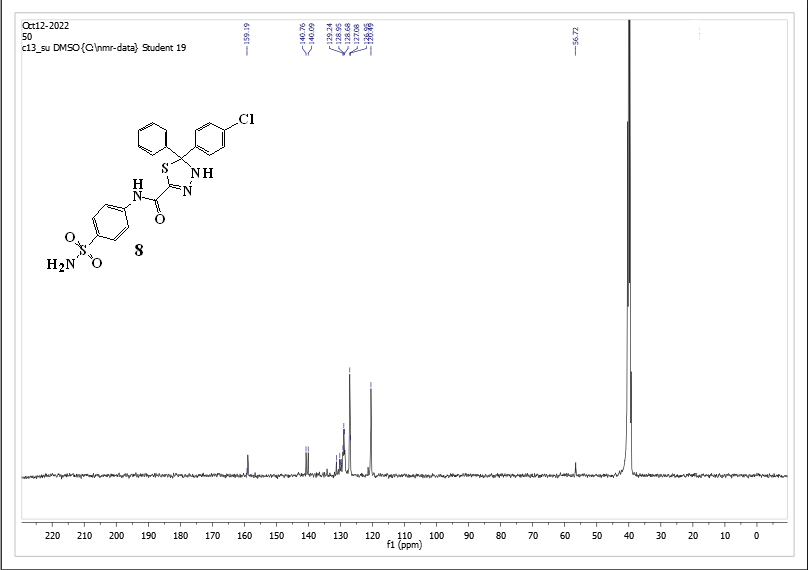


**Figure 25**: ^13^CNMR Spectrum of compound **8**


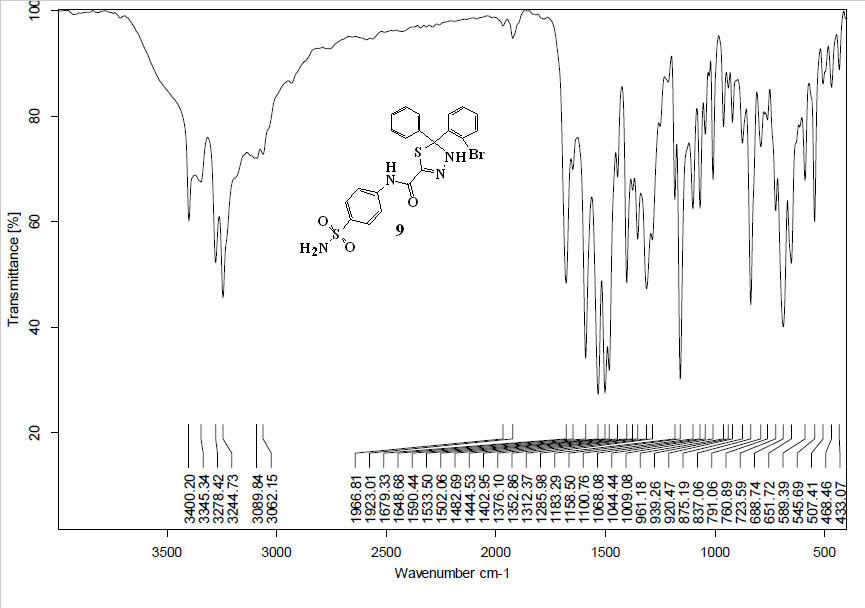


**Figure 26**: IR Spectrum of compound **9**


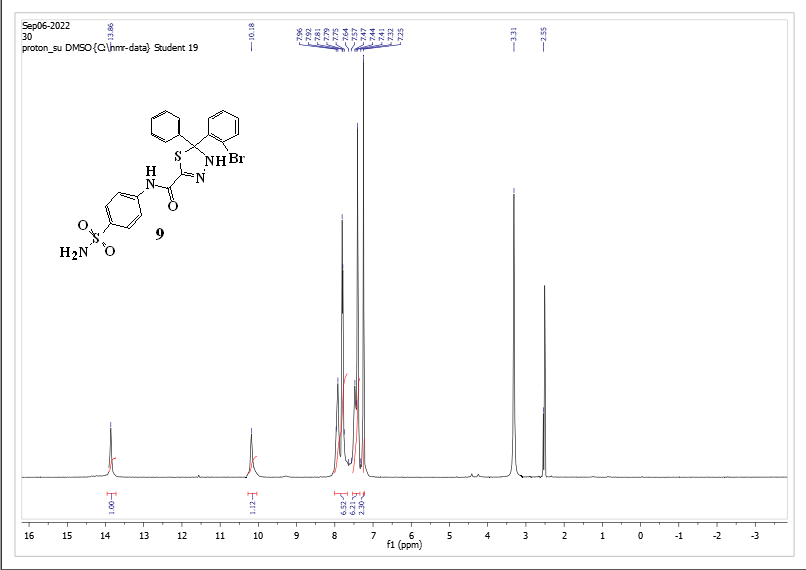


**Figure 27**: ^1^HNMR Spectrum of compound **9**


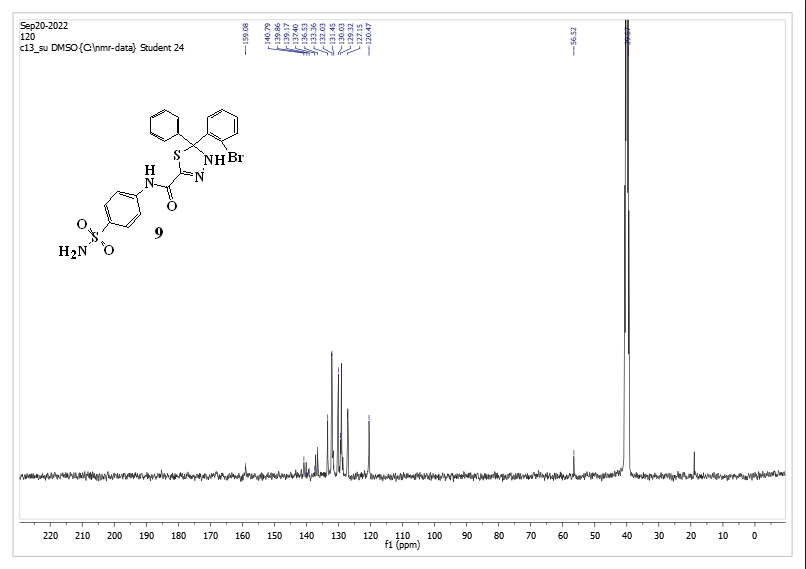


**Figure 28**: ^13^CNMR Spectrum of compound **9**


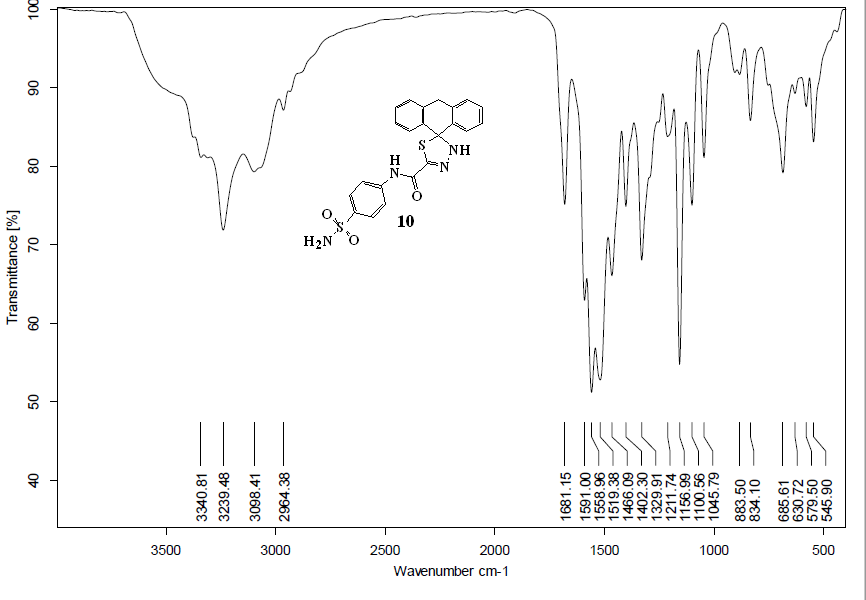


**Figure 29**: IR Spectrum of compound **10**


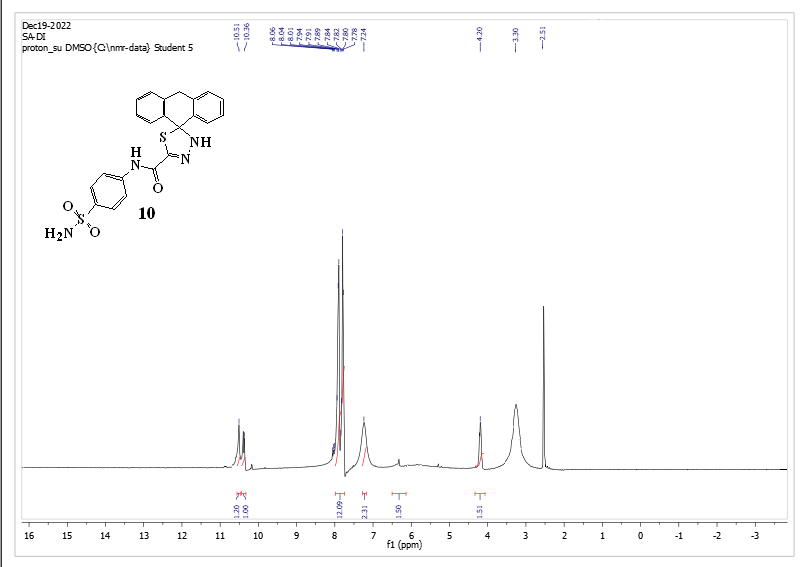


**Figure 30**: ^1^HNMR Spectrum of compound **10**


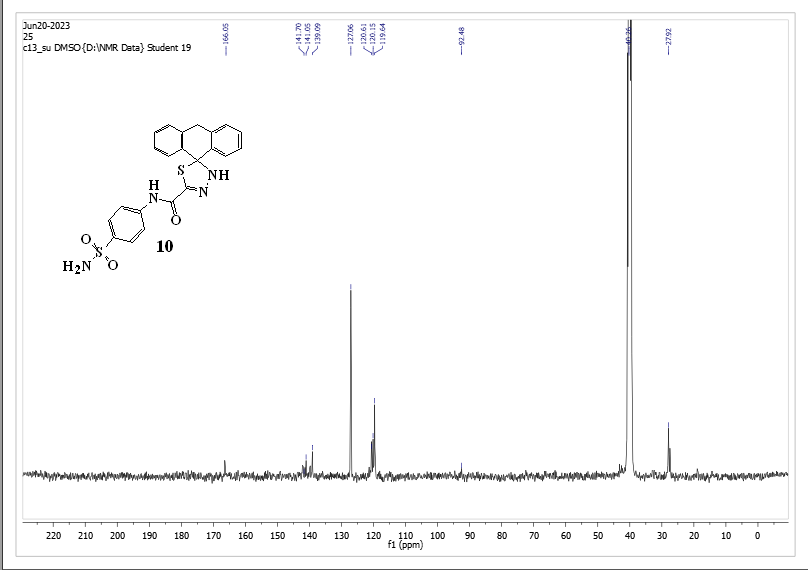


**Figure 31**: ^13^CNMR Spectrum of compound **10**


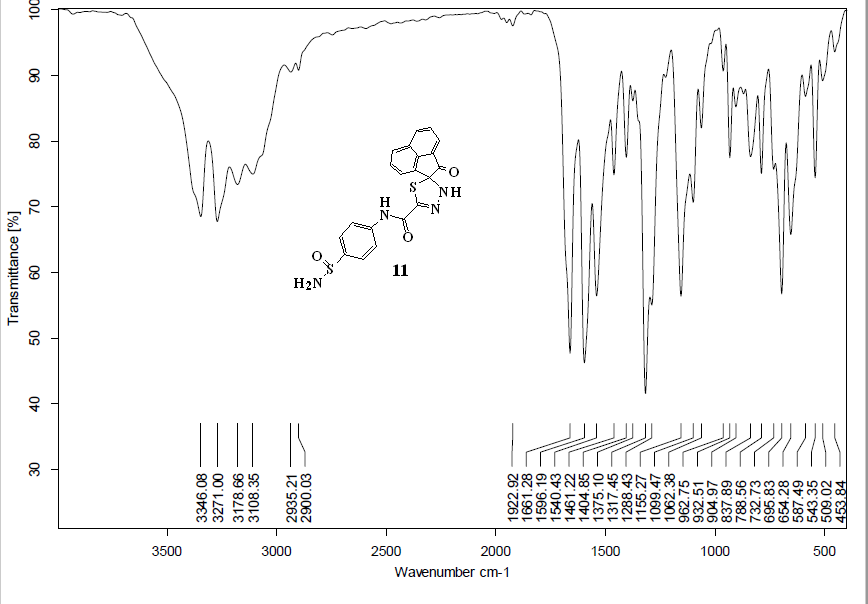


**Figure 32**: IR Spectrum of compound **11**


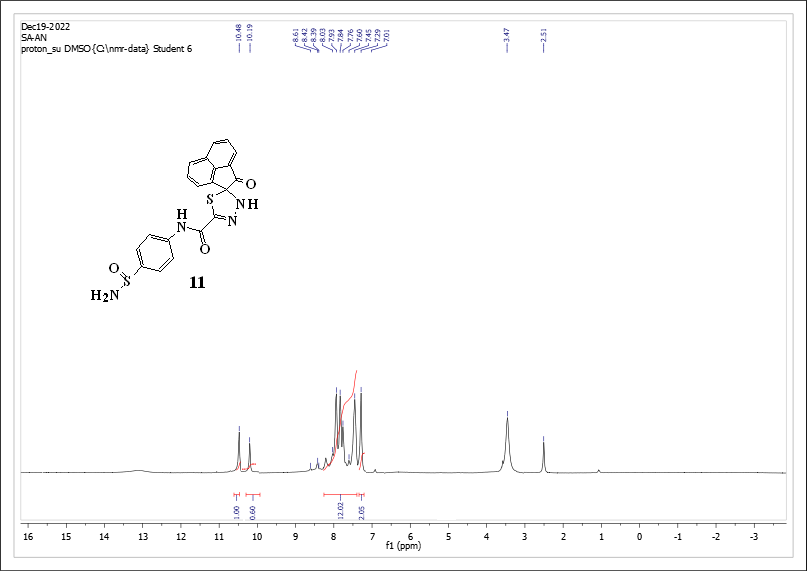


**Figure 33**: ^1^HNMR Spectrum of compound **11**


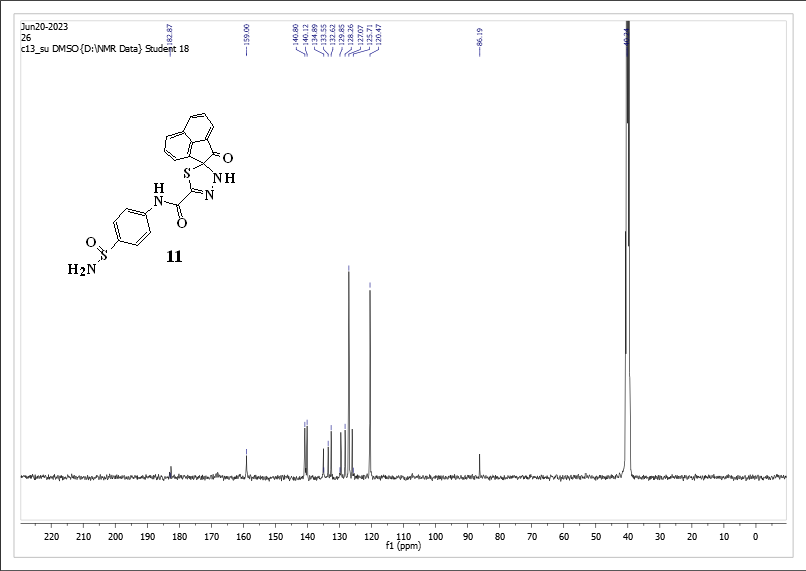


**Figure 34**: ^13^CNMR Spectrum of compound **11**


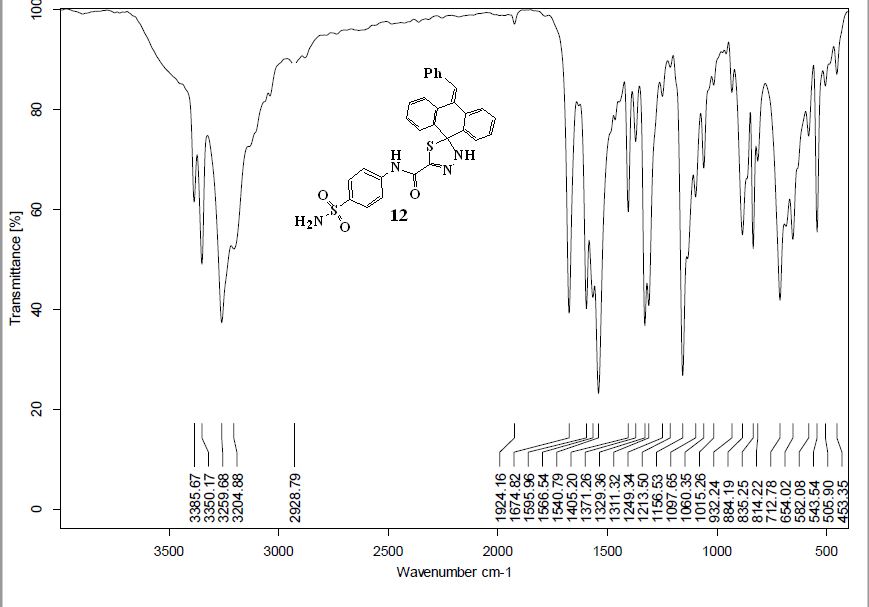


**Figure 35**: IR Spectrum of compound **12**


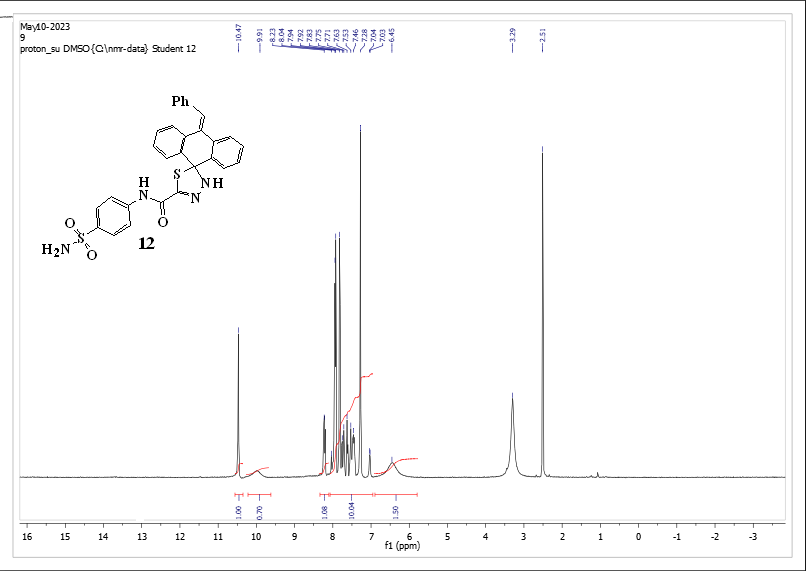


**Figure 36**: ^1^HNMR Spectrum of compound **12**


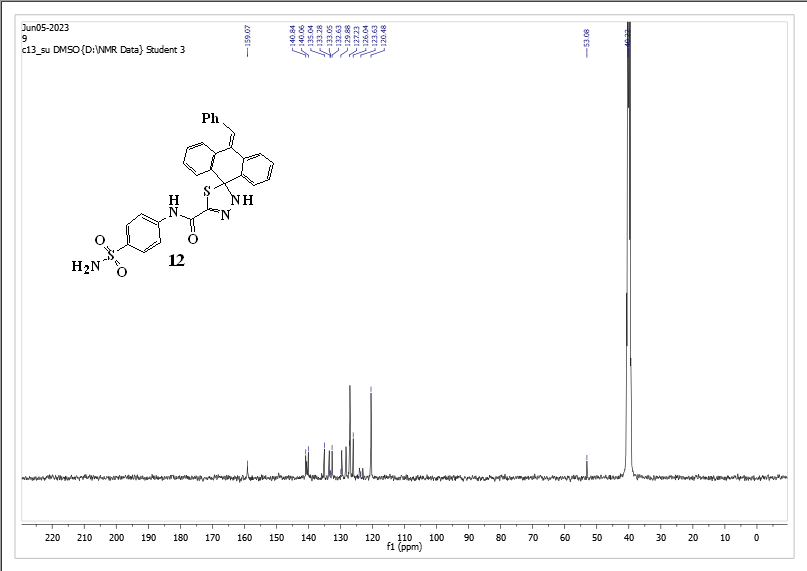


**Figure 37**: ^13^CNMR Spectrum of compound **12**

| **Table (S.1): Drug-Likeness and ADMET properties** | | | | | | | | | | | |
| --- | --- | --- | --- | --- | --- | --- | --- | --- | --- | --- | --- |
|  | **2** | **3** | **4** | **5** | **6** | **7** | **8** | **9** | **10** | **11** | **12** |
| **Physicochemical Property** | | | | | | | | | | | |
| Molecular Weight  (MW) | 354.08 | 340.07 | 368.1 | 368.1 | 396.09 | 403.04 | 472.04 | 515.99 | 450.08 | 422.05 | 538.11 |
| Volume | 319.224 | 301.928 | 336.52 | 336.52 | 359.97 | 354.501 | 431.132 | 435.205 | 424.661 | 390.069 | 526.63 |
| Density | 1.109 | 1.126 | 1.094 | 1.094 | 1.1 | 1.137 | 1.095 | 1.186 | 1.06 | 1.082 | 1.022 |
| nHA | 7 | 7 | 7 | 7 | 8 | 9 | 7 | 7 | 7 | 7 | 7 |
| nHD | 4 | 4 | 4 | 4 | 4 | 5 | 4 | 4 | 4 | 4 | 4 |
| nRot | 4 | 4 | 4 | 4 | 4 | 4 | 6 | 6 | 4 | 4 | 5 |
| nRing | 3 | 3 | 3 | 3 | 3 | 4 | 4 | 4 | 5 | 5 | 6 |
| MaxRing | 6 | 6 | 6 | 7 | 6 | 9 | 6 | 6 | 14 | 11 | 14 |
| nHet | 9 | 9 | 9 | 9 | 10 | 11 | 10 | 10 | 9 | 9 | 9 |
| fChar | 0 | 0 | 0 | 0 | 0 | 0 | 0 | 0 | 0 | 0 | 0 |
| nRig | 20 | 19 | 20 | 21 | 21 | 25 | 26 | 26 | 30 | 28 | 37 |
| Flexibility | 0.2 | 0.211 | 0.2 | 0.19 | 0.19 | 0.16 | 0.231 | 0.231 | 0.133 | 0.143 | 0.135 |
| Stereo Centers | 0 | 0 | 2 | 0 | 1 | 1 | 1 | 1 | 0 | 2 | 0 |
| TPSA | 113.65 | 113.65 | 113.65 | 113.65 | 130.72 | 142.75 | 113.65 | 113.65 | 113.65 | 113.65 | 113.65 |
| logS | -3.739 | -3.604 | -3.828 | -3.883 | -3.777 | -4.129 | -4.942 | -4.918 | -4.995 | -4.604 | -5.78 |
| logP | 1.894 | 1.404 | 2.048 | 2.347 | 1.441 | 1.155 | 3.41 | 3.316 | 3.304 | 2.097 | 4.416 |
| logD | 1.633 | 1.363 | 1.782 | 1.905 | 0.825 | 1.476 | 2.918 | 2.916 | 2.765 | 2.072 | 3.081 |
| **Medicinal Chemistry** | | | | | | | | | | | |
| QED | 0.762 | 0.765 | 0.753 | 0.755 | 0.708 | 0.595 | 0.526 | 0.479 | 0.568 | 0.6 | 0.348 |
| SAscore | 3.339 | 3.369 | 4.212 | 3.315 | 4.115 | 3.795 | 3.186 | 3.259 | 3.371 | 4.396 | 3.422 |
| Fsp3 | 0.429 | 0.385 | 0.467 | 0.467 | 0.438 | 0.062 | 0.048 | 0.048 | 0.091 | 0.05 | 0.034 |
| MCE-18 | 68.4 | 67.556 | 90.909 | 69.091 | 102 | 102.941 | 77.273 | 77.273 | 91.833 | 114.667 | 104.267 |
| NPscore | -1.168 | -1.205 | -0.97 | -1.119 | -0.772 | -1.016 | -1.245 | -1.08 | -0.929 | -0.543 | -0.878 |
| Lipinski Rule | Accepted | Accepted | Accepted | Accepted | Accepted | Accepted | Accepted | Accepted | Accepted | Accepted | Accepted |
| Pfizer Rule | Accepted | Accepted | Accepted | Accepted | Accepted | Accepted | Accepted | Accepted | Accepted | Accepted | Accepted |
| GSK Rule | Accepted | Accepted | Accepted | Accepted | Accepted | Rejected | Rejected | Rejected | Rejected | Rejected | Rejected |
| Golden Triangle | Accepted | Accepted | Accepted | Accepted | Accepted | Accepted | Accepted | Rejected | Accepted | Accepted | Rejected |
| PAINS | 0 alert(s) | 0 alert(s) | 0 alert(s) | 0 alert(s) | 0 alert(s) | 1 alert(s) | 0 alert(s) | 0 alert(s) | 0 alert(s) | 1 alert(s) | 0 alert(s) |
| ALARM NMR Rule | 3 alert(s) | 3 alert(s) | 3 alert(s) | 3 alert(s) | 4 alert(s) | 3 alert(s) | 3 alert(s) | 4 alert(s) | 3 alert(s) | 5 alert(s) | 3 alert(s) |
| BMS Rule | 0 alert(s) | 0 alert(s) | 0 alert(s) | 0 alert(s) | 0 alert(s) | 0 alert(s) | 0 alert(s) | 0 alert(s) | 0 alert(s) | 0 alert(s) | 0 alert(s) |
| Chelator Rule | 0 alert(s) | 0 alert(s) | 0 alert(s) | 0 alert(s) | 0 alert(s) | 0 alert(s) | 0 alert(s) | 0 alert(s) | 0 alert(s) | 0 alert(s) | 0 alert(s) |
| **Absorption** | | | | | | | | | | | |
| Caco-2 Permeability | -5.893 | -6.129 | -5.733 | -5.681 | -6.335 | -6.567 | -5.893 | -5.86 | -6.312 | -5.586 | -6.126 |
| MDCK Permeability | 6.30E-06 | 5.60E-06 | 5.70E-06 | 7.00E-06 | 1.10E-05 | 3.70E-06 | 1.20E-05 | 1.20E-05 | 5.40E-06 | 6.20E-06 | 1.10E-05 |
| Pgp-inhibitor | --- | --- | --- | --- | --- | --- | --- | --- | - | --- | - |
| Pgp-substrate | --- | --- | --- | --- | --- | --- | --- | --- | --- | --- | --- |
| HIA | --- | --- | --- | --- | --- | --- | + | --- | -- | --- | - |
| F20% | --- | --- | --- | --- | --- | --- | --- | --- | --- | --- | --- |
| F30% | --- | --- | -- | --- | --- | --- | --- | --- | --- | --- | --- |
| **Distribution** | | | | | | | | | | | |
| PPB | 91.31% | 87.43% | 91.26% | 93.74% | 59.37% | 94.41% | 97.72% | 97.82% | 97.13% | 97.26% | 98.39% |
| VD | 0.474 | 0.476 | 0.468 | 0.463 | 0.573 | 0.568 | 0.674 | 0.704 | 0.355 | 0.473 | 0.599 |
| BBB Penetration | -- | -- | -- | -- | --- | --- | --- | --- | --- | --- | --- |
| Fu | 11.23% | 17.06% | 14.04% | 7.01% | 39.75% | 5.94% | 2.18% | 3.78% | 2.03% | 1.86% | 0.77% |
| **Metabolism** | | | | | | | | | | | |
| CYP1A2 inhibitor | - | - | ++ | + | --- | -- | - | - | -- | +++ | - |
| CYP1A2 substrate | ++ | +++ | + | ++ | + | + | -- | -- | - | + | + |
| CYP2C19 inhibitor | -- | -- | -- | -- | -- | --- | +++ | +++ | ++ | + | +++ |
| CYP2C19 substrate | -- | --- | -- | -- | --- | -- | - | + | + | --- | - |
| CYP2C9 inhibitor | + | - | ++ | + | + | ++ | +++ | +++ | +++ | +++ | +++ |
| CYP2C9 substrate | ++ | ++ | ++ | +++ | ++ | +++ | +++ | +++ | +++ | +++ | +++ |
| CYP2D6 inhibitor | + | - | + | + | --- | --- | ++ | + | + | - | - |
| CYP2D6 substrate | -- | -- | -- | -- | -- | -- | -- | -- | -- | -- | -- |
| CYP3A4 inhibitor | -- | -- | -- | -- | -- | -- | + | ++ | ++ | ++ | ++ |
| CYP3A4 substrate | -- | -- | -- | -- | - | +++ | +++ | +++ | +++ | ++ | +++ |
| **Excretion** | | | | | | | | | | | |
| CL | 1.196 | 1.233 | 1.235 | 1.168 | 1.353 | 1.06 | 1.11 | 1.257 | 1.324 | 1.266 | 1.224 |
| T1/2 | 0.142 | 0.181 | 0.287 | 0.111 | 0.376 | 0.152 | 0.118 | 0.193 | 0.039 | 0.154 | 0.009 |
| **Toxicity** | | | | | | | | | | | |
| hERG Blockers | --- | --- | --- | --- | --- | --- | --- | --- | --- | --- | --- |
| H-HT | - | + | - | - | ++ | - | -- | -- | -- | - | - |
| DILI | +++ | +++ | +++ | +++ | +++ | +++ | +++ | +++ | +++ | +++ | +++ |
| AMES Toxicity | --- | --- | -- | --- | --- | -- | --- | --- | - | +++ | + |
| Rat Oral Acute Toxicity | --- | --- | -- | --- | -- | -- | + | + | + | -- | + |
| FDAMDD | +++ | ++ | +++ | +++ | +++ | ++ | +++ | +++ | +++ | ++ | +++ |
| Skin Sensitization | -- | -- | --- | -- | --- | --- | --- | --- | --- | -- | --- |
| Carcinogencity | ++ | ++ | ++ | ++ | +++ | ++ | ++ | ++ | ++ | +++ | + |
| Eye Corrosion | --- | --- | --- | --- | --- | --- | --- | --- | --- | --- | --- |
| Eye Irritation | -- | -- | --- | -- | --- | --- | --- | --- | -- | --- | --- |
| Respiratory Toxicity | ++ | + | - | ++ | --- | - | -- | -- | --- | ++ | -- |
| Environmental Toxicity |  |  |  |  |  |  |  |  |  |  |  |
| Bioconcentration Factors | 0.485 | 0.461 | 0.565 | 0.516 | 0.252 | 0.54 | 0.875 | 0.776 | 1.046 | 1.008 | 1.528 |
| IGC50 | 3.845 | 3.606 | 3.863 | 4.119 | 2.501 | 3.776 | 4.566 | 4.61 | 4.935 | 4.896 | 5.027 |
| LC50FM | 4.363 | 4.193 | 4.735 | 4.533 | 2.29 | 4.207 | 5.396 | 5.495 | 5.465 | 5.712 | 5.774 |
| LC50DM | 4.557 | 4.489 | 4.96 | 4.624 | 3.82 | 4.673 | 5.542 | 5.652 | 5.479 | 5.817 | 5.935 |
| Tox21 Pathway |  |  |  |  |  |  |  |  |  |  |  |
| NR-AR | --- | --- | --- | --- | --- | --- | --- | --- | --- | --- | --- |
| NR-AR-LBD | --- | --- | --- | --- | --- | --- | --- | --- | --- | --- | --- |
| NR-AhR | -- | -- | -- | - | --- | -- | - | - | ++ | +++ | ++ |
| NR-Aromatase | --- | --- | --- | --- | --- | --- | --- | --- | --- | --- | --- |
| NR-ER | - | - | -- | - | -- | -- | + | - | - | ++ | ++ |
| NR-ER-LBD | --- | --- | --- | --- | --- | --- | --- | --- | --- | --- | --- |
| NR-PPAR-gamma | --- | --- | --- | --- | --- | --- | --- | --- | - | --- | + |
| SR-ARE | ++ | ++ | ++ | ++ | ++ | + | ++ | ++ | ++ | +++ | +++ |
| SR-ATAD5 | --- | --- | --- | --- | --- | --- | --- | --- | --- | --- | --- |
| SR-HSE | --- | --- | --- | --- | --- | --- | --- | --- | --- | --- | --- |
| SR-MMP | + | + | + | + | + | ++ | ++ | ++ | +++ | ++ | +++ |
| SR-p53 | -- | -- | -- | - | -- | -- | -- | -- | - | ++ | - |
| Toxicophore Rules |  |  |  |  |  |  |  |  |  |  |  |
| Acute Toxicity Rule | 0 alert(s) | 0 alert(s) | 0 alert(s) | 0 alert(s) | 0 alert(s) | 0 alert(s) | 0 alert(s) | 0 alert(s) | 0 alert(s) | 0 alert(s) | 0 alert(s) |
| Genotoxic Carcinogenicity Rule | 1 alert(s) | 1 alert(s) | 1 alert(s) | 1 alert(s) | 1 alert(s) | 1 alert(s) | 1 alert(s) | 1 alert(s) | 1 alert(s) | 5 alert(s) | 1 alert(s) |
| NonGenotoxic Carcinogenicity Rule | 0 alert(s) | 0 alert(s) | 0 alert(s) | 0 alert(s) | 0 alert(s) | 0 alert(s) | 1 alert(s) | 1 alert(s) | 0 alert(s) | 1 alert(s) | 0 alert(s) |
| Skin Sensitization Rule | 4 alert(s) | 4 alert(s) | 4 alert(s) | 4 alert(s) | 5 alert(s) | 5 alert(s) | 5 alert(s) | 5 alert(s) | 5 alert(s) | 6 alert(s) | 6 alert(s) |
| Aquatic Toxicity Rule | 0 alert(s) | 0 alert(s) | 0 alert(s) | 0 alert(s) | 0 alert(s) | 0 alert(s) | 1 alert(s) | 1 alert(s) | 0 alert(s) | 1 alert(s) | 0 alert(s) |
| NonBiodegradable Rule | 0 alert(s) | 0 alert(s) | 0 alert(s) | 0 alert(s) | 1 alert(s) | 0 alert(s) | 1 alert(s) | 0 alert(s) | 0 alert(s) | 0 alert(s) | 0 alert(s) |
| SureChEMBL Rule | 0 alert(s) | 0 alert(s) | 0 alert(s) | 0 alert(s) | 0 alert(s) | 0 alert(s) | 0 alert(s) | 0 alert(s) | 0 alert(s) | 0 alert(s) | 0 alert(s) |
| FAF-Drugs4 Rule | 1 alert(s) | 1 alert(s) | 1 alert(s) | 1 alert(s) | 1 alert(s) | 1 alert(s) | 1 alert(s) | 1 alert(s) | 1 alert(s) | 1 alert(s) | 1 alert(s) |
|  |  |  |  |  |  |  |  |  |  |  |  |

| (a) | 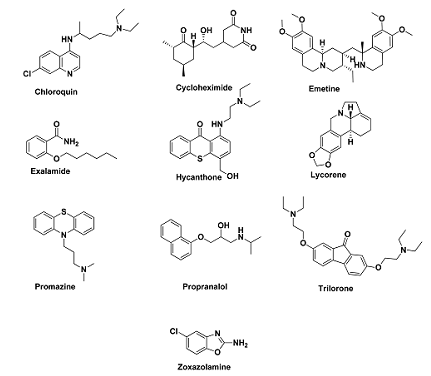 |
| --- | --- |
| (b) | 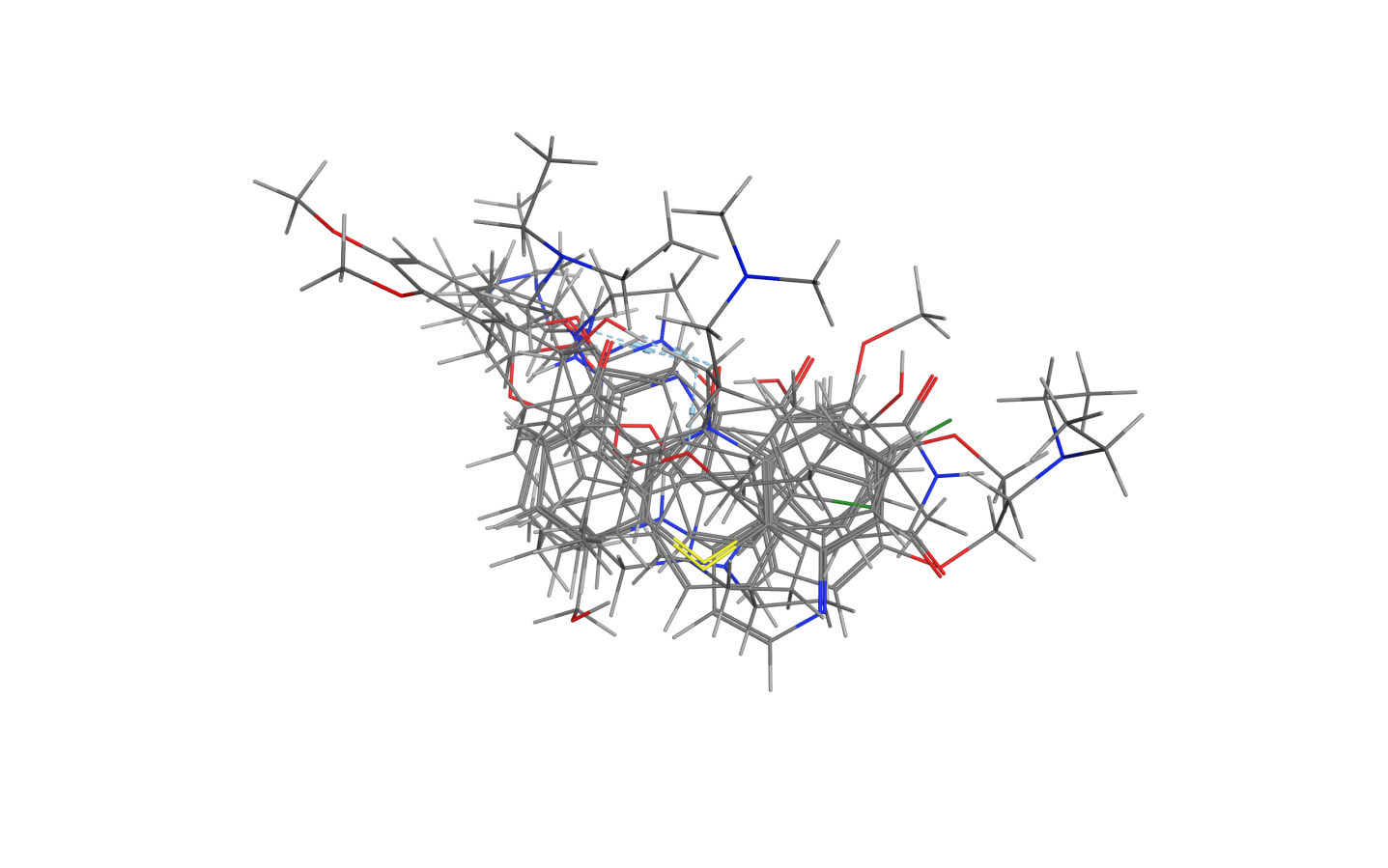 |
| (c) | 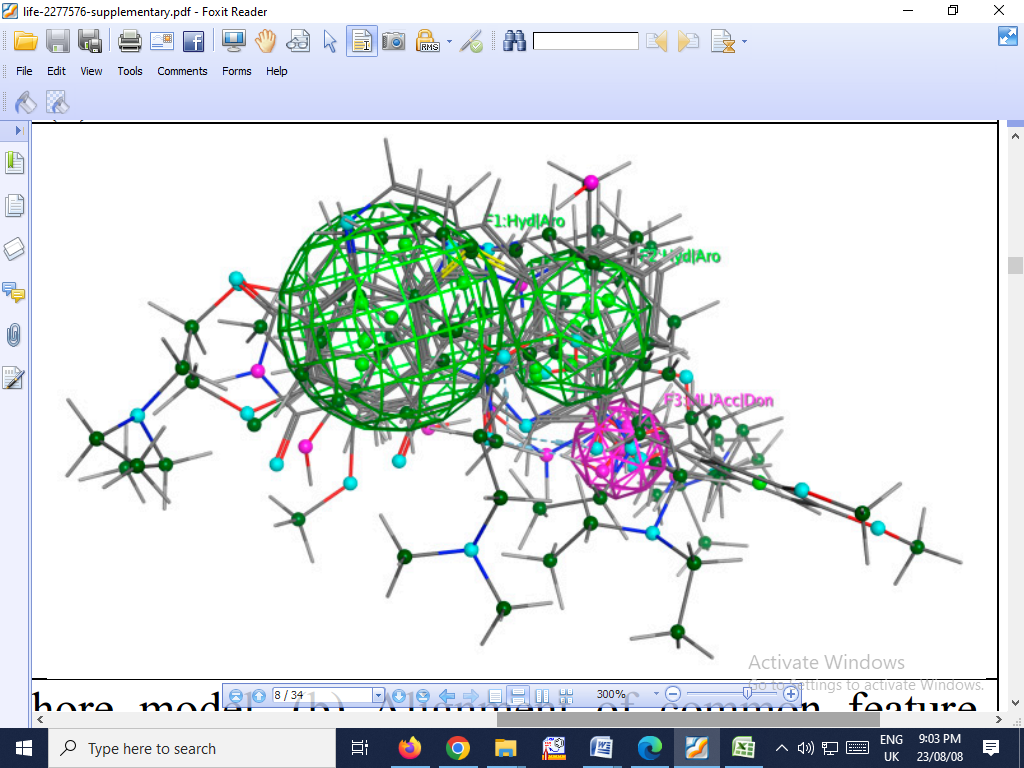 |
| Figure (S38): (a) Structures of training set compounds, (b) Alignment of ten approved FDA drugs (Chloroquine, Cycloheximide, Emetine, Exalamide, Hycanthone, Lycorine, Promazin, Propranalol, Trilorene, and Zoxazolamine) from the training set, carbon atoms are in gray, oxygen atoms are in red, nitrogen atom in blue, (c): Alignment of common feature pharmacophore model with the training set;. | |

| Chloroquine (RMSD = 0.0126) | Cycloheximide (RMSD = 0.0841) |
| --- | --- |
| 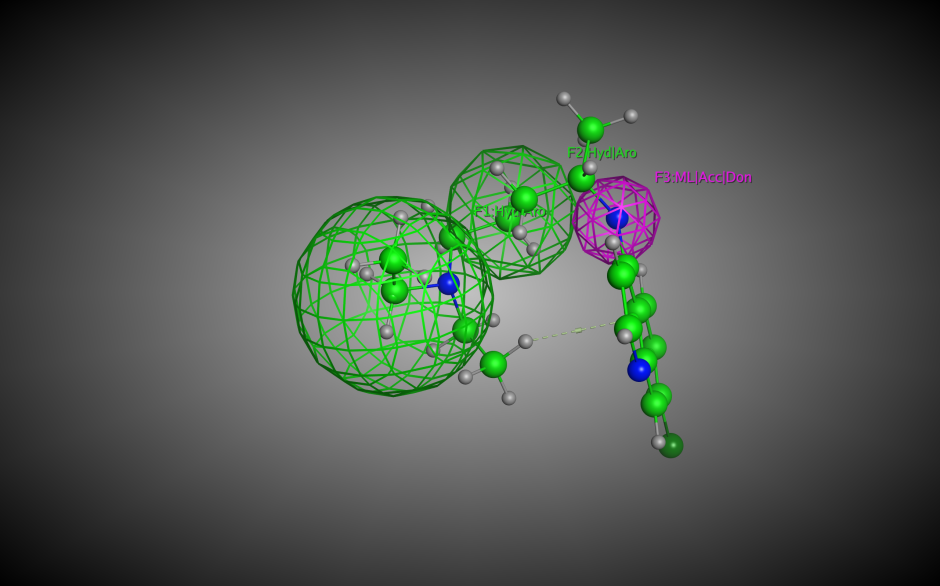 | 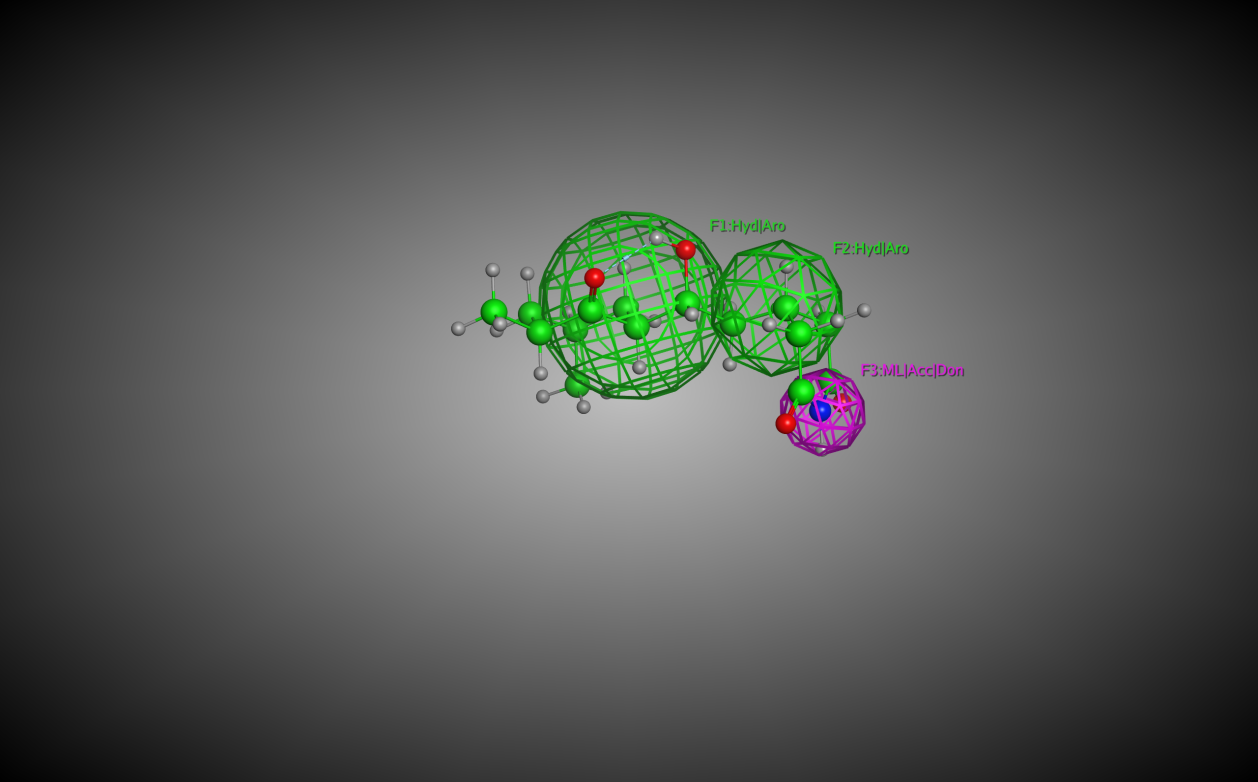 |
| Emetine (RMSD =0.0226) | Exalamide (RMSD =0.0513) |
| 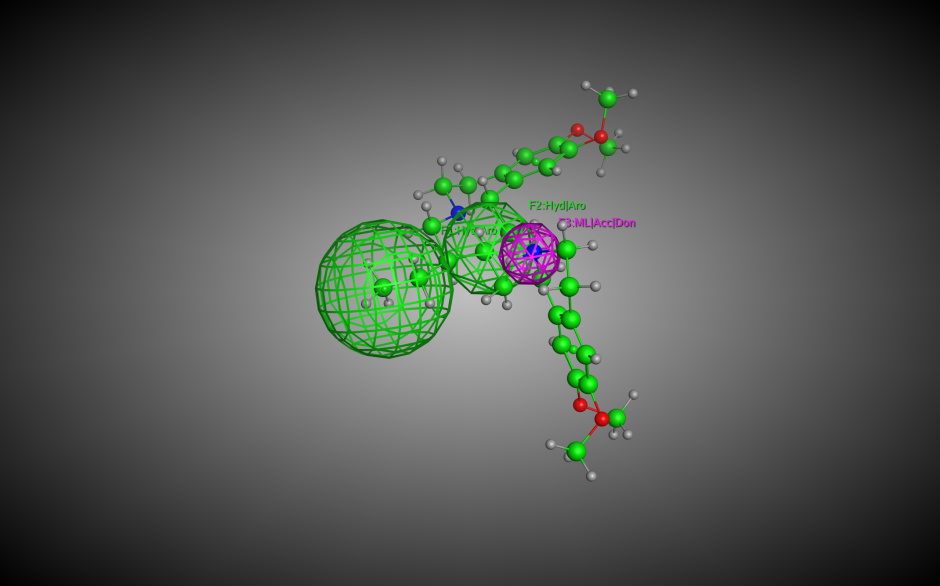 | 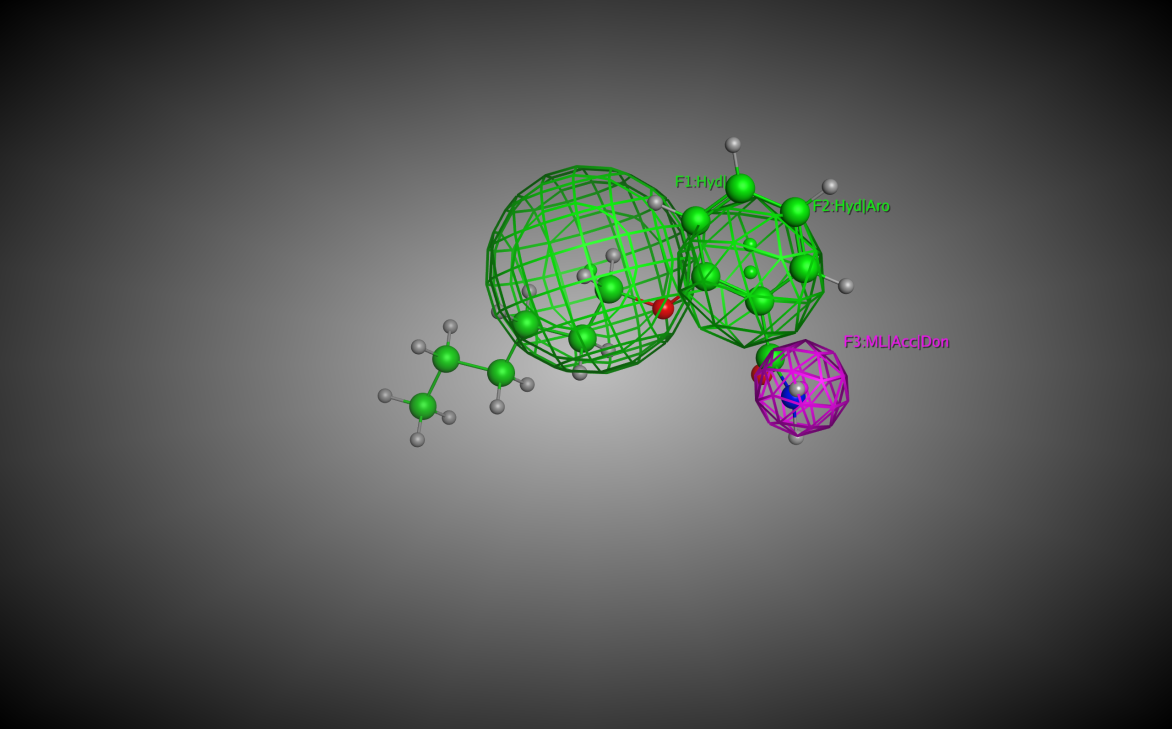 |
| Hycanthone (RMSD =0.0343) | Lycorine (RMSD =0.0180) |
| 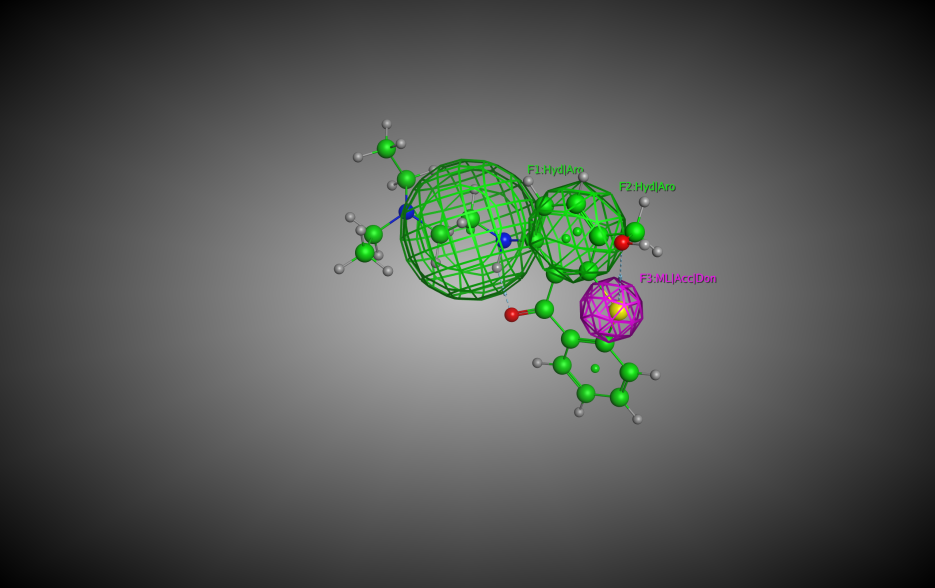 | 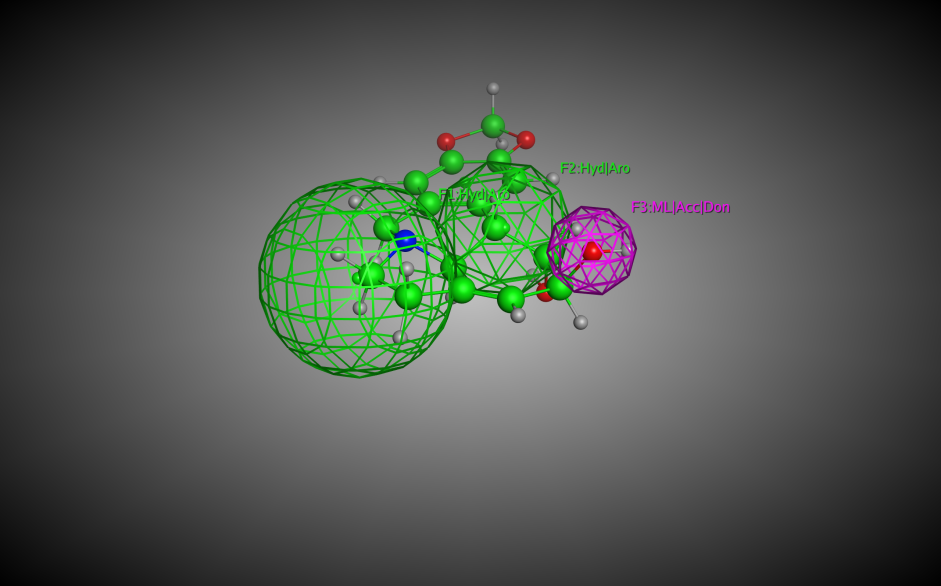 |
| Promazin (RMSD =0.0930) | Propranalol (RMSD =0.0219) |
| 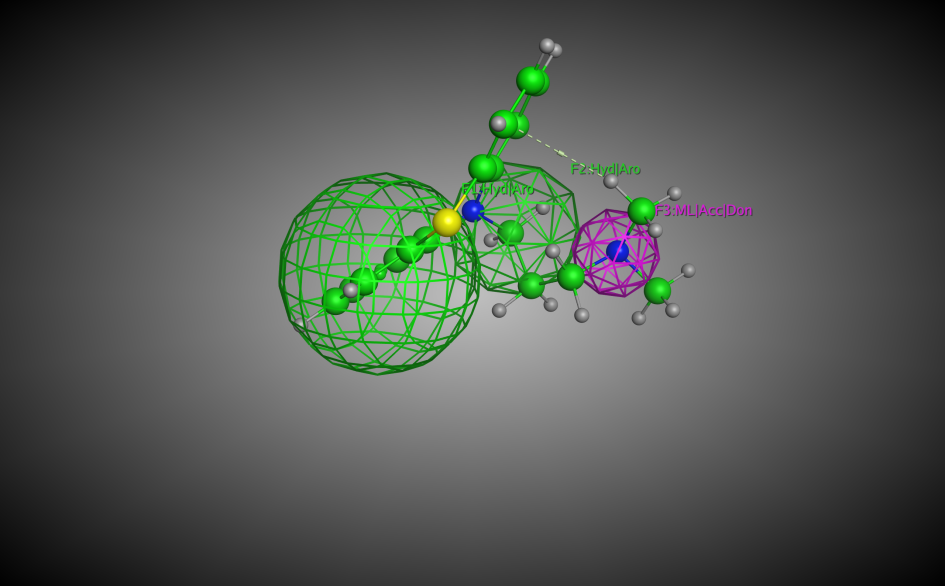 | 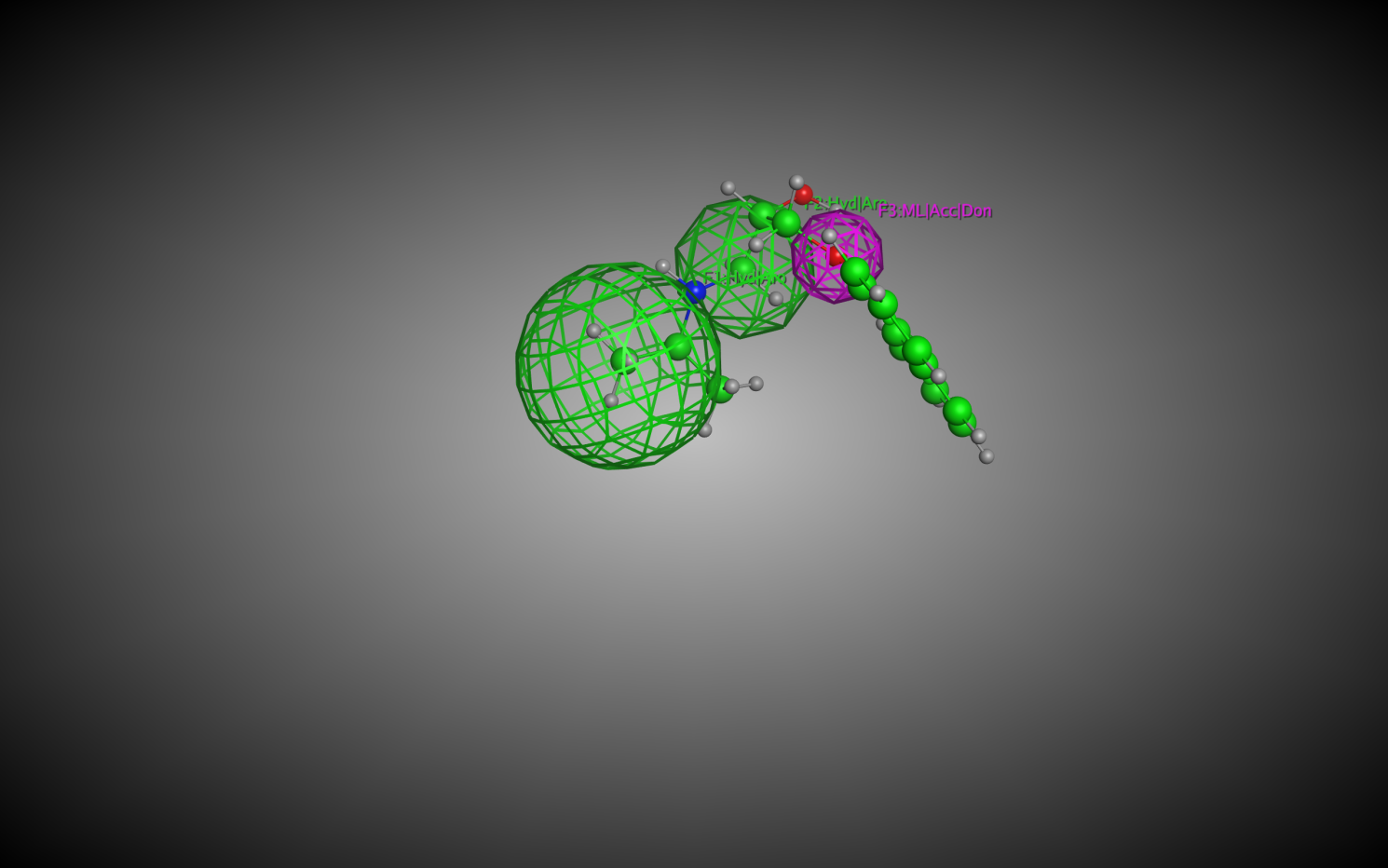 |
| Trilorene (RMSD =0.0316) | Zoxazolamine (RMSD =0.0405) |
| 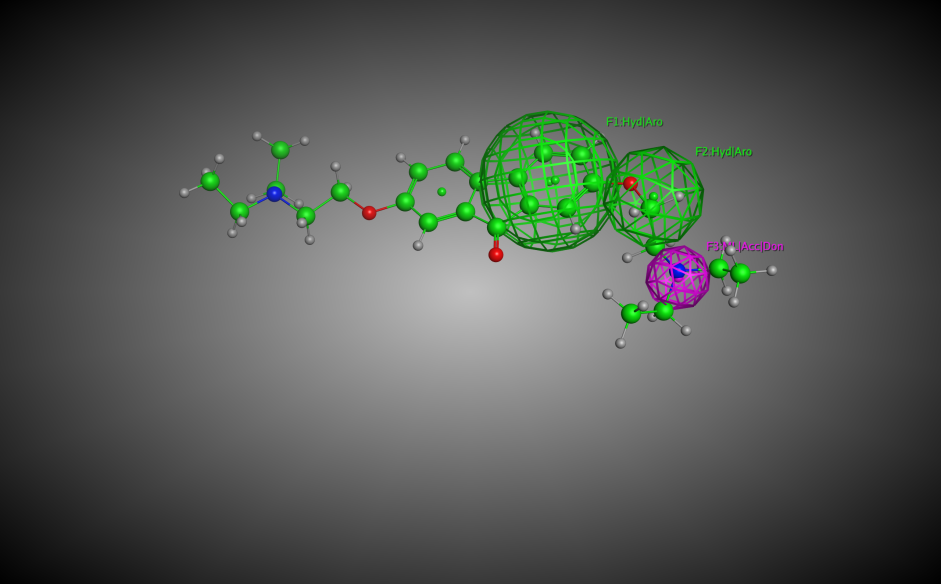 | 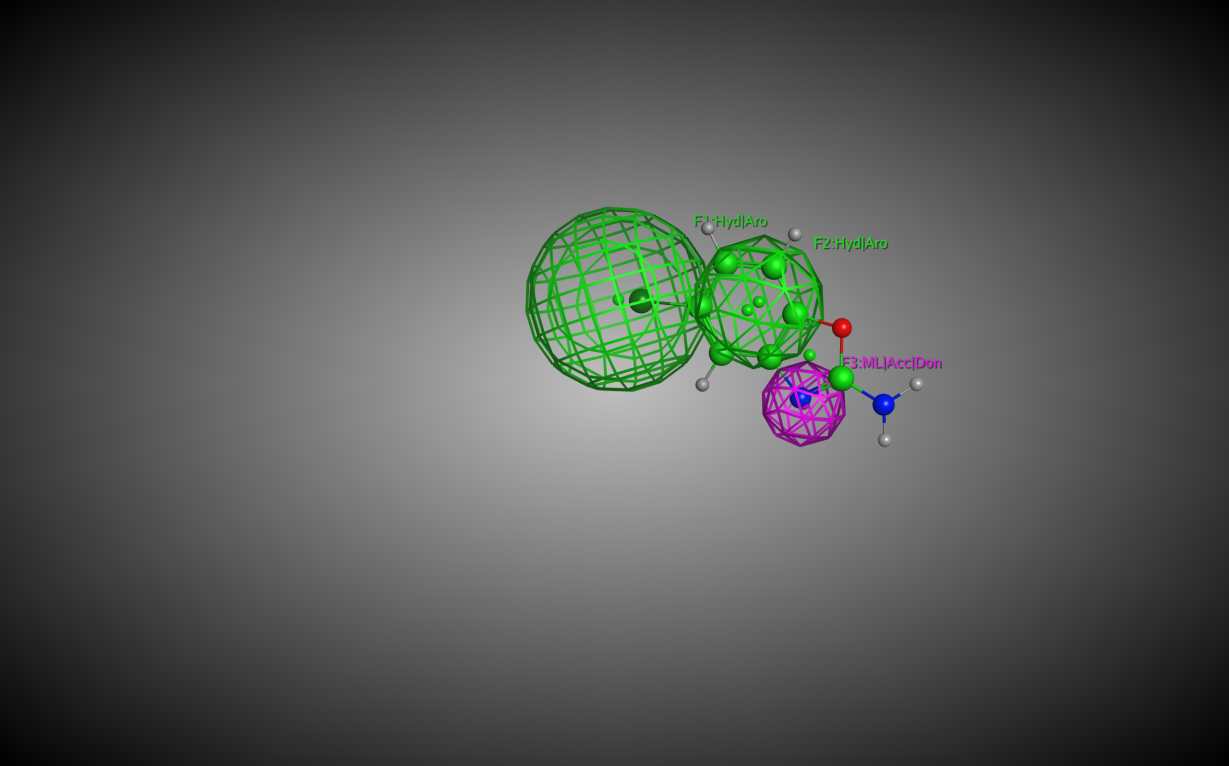 |
| Nirmatrelvir (RMSD =0.079) | Ritonavir (RMSD =0.0315) |
| 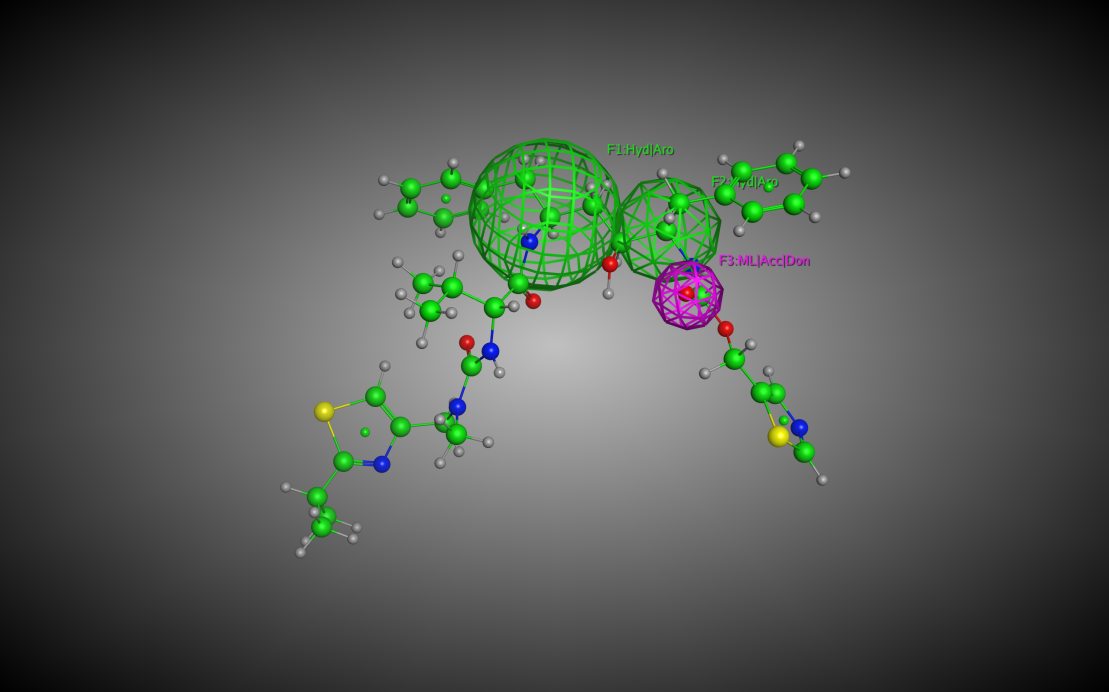 | 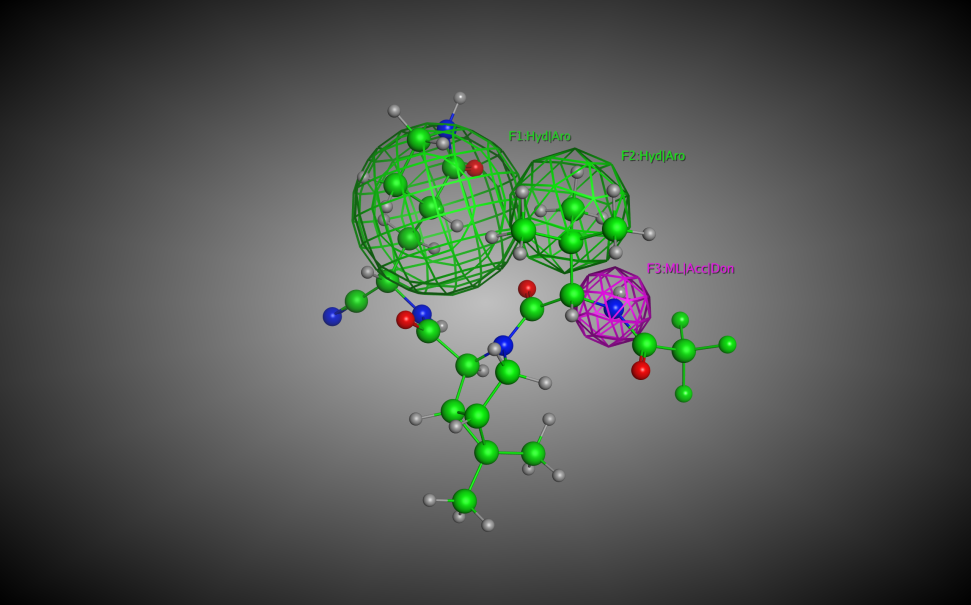 |
| Figure (S39): The validation of the pharmacophore model using training set: Chloroquine, Cycloheximide, Emetine, Exalamide, Hycanthone, Lycorine, Promazin, Propranalol, Trilorene, and Zoxazolamine, and two active Nirmatrelvir and Ritonavir drugs with their (RMSD). | |

| (a) | (b) |
| --- | --- |
| 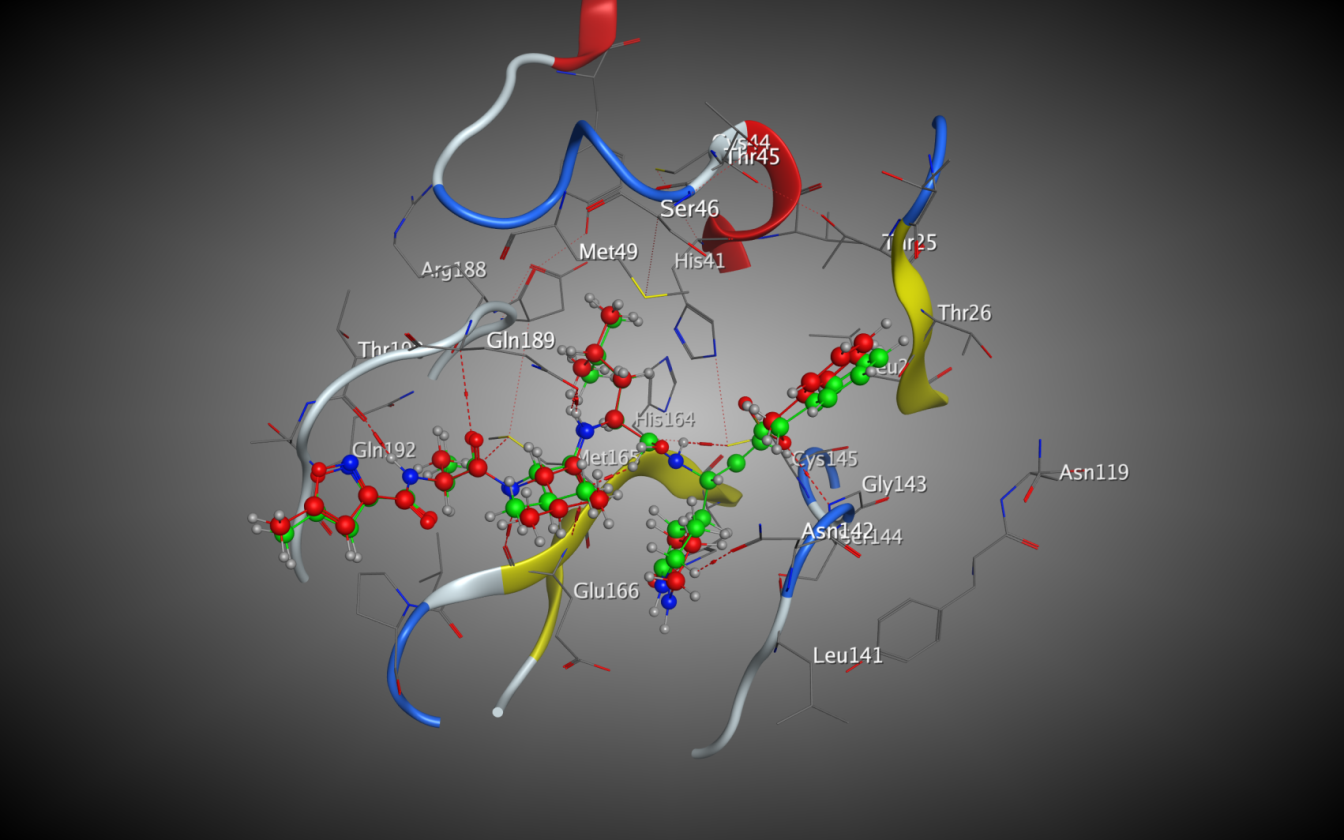 | 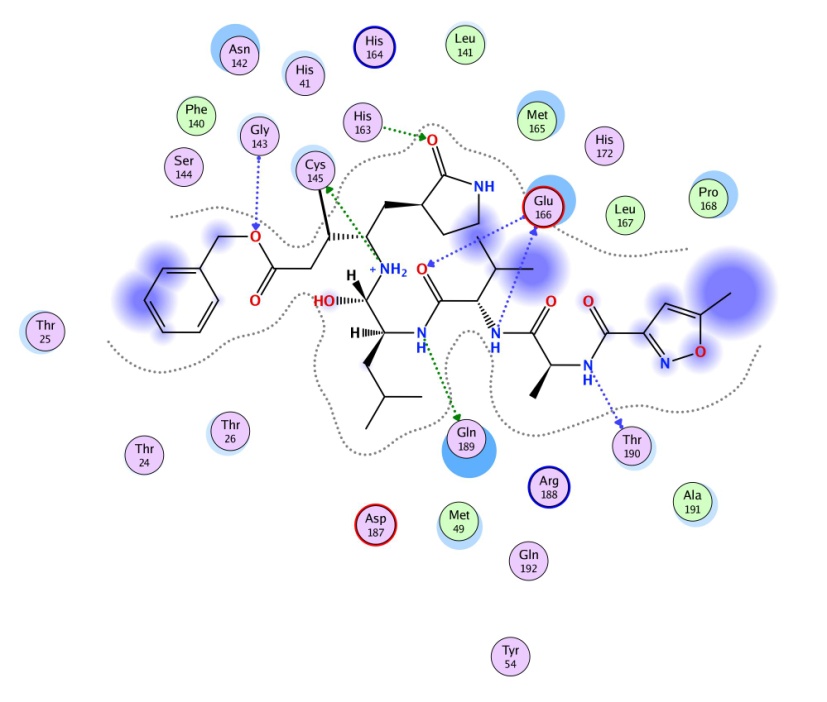 |
| Figure. (S40): (a) 3D representation of the superimposition of the native ligand (red) and the docking pose (green) of the ligand in 6lu7 binding site, (b): 2D interaction of the re-docked ligand with residue amino acids. | |
